# A Phylogeny-Regularized Sparse Regression Model for Predictive Modeling of Microbial Community Data

**DOI:** 10.3389/fmicb.2018.03112

**Published:** 2018-12-19

**Authors:** Jian Xiao, Li Chen, Yue Yu, Xianyang Zhang, Jun Chen

**Affiliations:** ^1^Division of Biomedical Statistics and Informatics, Center for Individualized Medicine, Mayo Clinic Rochester, MN, United States; ^2^School of Statistics and Mathematics Zhongnan University of Economics and Law, Wuhan, China; ^3^Department of Health Outcomes Research and Policy, Harrison School of Pharmacy, Auburn University Auburn, AL, United States; ^4^Department of Statistics, Texas A&M University College Station, TX, United States

**Keywords:** microbiome, phylogenetic tree, sparse generalized linear model, predictive model, statistical modeling, high-dimenisonal statistics

## Abstract

Fueled by technological advancement, there has been a surge of human microbiome studies surveying the microbial communities associated with the human body and their links with health and disease. As a complement to the human genome, the human microbiome holds great potential for precision medicine. Efficient predictive models based on microbiome data could be potentially used in various clinical applications such as disease diagnosis, patient stratification and drug response prediction. One important characteristic of the microbial community data is the phylogenetic tree that relates all the microbial taxa based on their evolutionary history. The phylogenetic tree is an informative prior for more efficient prediction since the microbial community changes are usually not randomly distributed on the tree but tend to occur in clades at varying phylogenetic depths (*clustered signal*). Although community-wide changes are possible for some conditions, it is also likely that the community changes are only associated with a small subset of “marker” taxa (*sparse signal*). Unfortunately, predictive models of microbial community data taking into account both the sparsity and the tree structure remain under-developed. In this paper, we propose a predictive framework to exploit *sparse* and *clustered* microbiome signals using a phylogeny-regularized sparse regression model. Our approach is motivated by evolutionary theory, where a natural correlation structure among microbial taxa exists according to the phylogenetic relationship. A novel phylogeny-based smoothness penalty is proposed to smooth the coefficients of the microbial taxa with respect to the phylogenetic tree. Using simulated and real datasets, we show that our method achieves better prediction performance than competing sparse regression methods for sparse and clustered microbiome signals.

## 1. Introduction

The human microbial community (a.k.a., microbiota) is the collection of microorganisms associated with the human body. These microorganisms, their genomes, and the environment they reside in are collectively known as the human “microbiome.” The human microbiome plays a critical role in health and disease (Cho and Blaser, 2012). For instance, the human gut microbiome aids the digestive system with inaccessible nutrients, synthesizes beneficial nutrients and protects us against pathogens. An abnormal microbiome has been implicated in many human diseases including various cancer types (Ahn et al., 2013; Bultman, 2014; Walther-Antonio et al., 2016; Peters et al., 2017). Dysbiosis of the microbiome has been observed in obesity, type II diabetes, rheumatoid arthritis and multiple sclerosis (Turnbaugh et al., 2009; Kinross et al., 2011; Honda and Littman, 2012; Pflughoeft and Versalovic, 2012; Qin et al., 2012; Chen et al., 2016; Jangi et al., 2016). Therefore, the human microbiome holds great potential for various clinical applications such as disease diagnosis, patient stratification and drug response prediction. Building up an efficient microbiome-based predictor could thus empower microbiome-based precision medicine (Kashyap et al., 2017).

Advances in low-cost, high-throughput DNA sequencing technologies such as Illumina Solexa sequencing has enabled researchers to study the microbiome composition by directly sequencing the microbial DNA. Two main approaches have been employed to sequence the microbiome: gene-targeted sequencing and shotgun metagenomic sequencing (Kuczynski et al., 2011). Compared to the shotgun metagenomic sequencing, where all microbial DNA is sequenced, the gene-targeted approach only sequences a “fingerprint” region of a “molecular clock” gene such as the 16S rRNA gene in the bacteria. Although the shotgun metagenomic sequencing provides more biological information, the targeted approach is still the dominant approach for large-scale microbiome studies due to its lower cost and high scalability (McDonald et al., 2018). In the targeted sequencing, standard practices involve clustering the sequencing reads into operational taxonomic units (OTUs) or amplicon sequence variants (ASVs) based on their sequence similarities (Schloss et al., 2009; Caporaso et al., 2010, 2012; Chen et al., 2013b, 2017; Edgar, 2013; Rideout et al., 2014; Callahan et al., 2016; Amir et al., 2017). A taxonomic lineage is further assigned to each OTU/ASV by comparing their sequence to existing 16S rRNA gene databases. Finally, a phylogenetic tree, which characterizes the evolutionary relationships among OTUs/ASVs, is constructed based on their sequence divergences (Price et al., 2010). For shotgun metagenomic sequencing, a phylogenetic tree can also be constructed based on the reference genomes of the detected species (Kembel et al., 2011). As a result, a typical microbiome sequencing study is usually summarized as a table of the read counts of the detected OTUs/ASVs/Species, together with a phylogenetic tree, reflecting the community structure and composition of the studied microbiome. For simplicity, hereafter, we use the term “OTU” to stand for the basic taxonomic units (e.g., OTU, ASV, species, taxa) from any sequencing experiment/bioinformatics pipeline. Compared to other types of omics sequencing data, one important characteristic of microbiome sequencing data (microbial community data) is the phylogenetic tree that relates all the OTUs. The phylogenetic tree provides prior knowledge about how the OTUs are evolutionarily related. Related OTUs, which usually share similar biological functions, are more likely to be simultaneously associated with the outcome, forming “clustered signals” at varying phylogenetic depths (Garcia et al., 2014; Martiny et al., 2015). Therefore, the phylogeny creates linkages among OTUs and induces a grouping structure, allowing more efficient linkage between the OTUs and the phenotype. As the microbial community data moves into even higher resolutions such as strain-level resolution (Mallick et al., 2017; Edgar, 2018), the phylogenetic relationship becomes even more important for OTU data analysis. Clearly, it is not sensible to treat OTUs with only 1% sequence divergence in the same way as the OTUs with more than 10% sequence divergence. Indeed, incorporating the tree structure has proven to make the analyses more efficient and robust for various statistical tasks ranging from ordination to microbiome-wide multiple testing (Purdom, 2011; Chen et al., 2012, 2013a; Evans and Matsen, 2012; Wang and Zhao, 2017; Xiao et al., 2017).

One important task for microbiome analysis is to predict the phenotype/outcome (either quantitative or qualitative) based on the features of the underlying microbial community (relative abundances of the OTUs and their phylogeny). This process is also known as predictive modeling or supervised learning in machine learning literature, where we try to derive some function from the training data that can be used to predict the outcome of future data, and to learn which features (i.e., OTUs) are predictive of the outcome. For clinical applications, the outcome includes disease state, treatment response, and drug toxicity. To enable prediction based on microbial community data, general-purpose predictive methods have been applied (Knights et al., 2011; Statnikov et al., 2013; Pasolli et al., 2016). These methods include classical machine learning methods (e.g., Random Forest and Support Vector Machine) and modern regression methods for high-dimensional data [e.g., Lasso (Tibshirani, 1996), MCP (Zhang, 2010), and Elastic Net (Zou and Hastie, 2005)], focusing on modeling the nonlinear relationship between the outcome and the microbiome as well as selecting the most predictive OTUs for better interpretation. However, these methods do not fully exploit the information in the microbiome data, particularly the phylogenetic relationship among OTUs. The phylogenetic tree is an informative prior since the microbial community changes are usually not randomly distributed but tend to occur in clades at varying phylogenetic depths (*clustered signal*). In other words, the phylogenetic structure offers a biologically motivated grouping structure, through which we can aggregate sparse OTU data to enrich signals and achieve better predictive performance. The objective of the proposed study is thus to provide a data-adaptive approach to use the tree structure when constructing the predictive model, i.e., let the data determine how much phylogenetic information and what level of phylogenetic depth we should use to achieve optimal performance. The inputs of our method are the OTU count table, the phylogenetic tree of the OTUs and the outcome measurements, and the outputs are the selected OTUs and the predictive function based on their abundances.

Many previous attempts have been made to incorporate the tree information into prediction, particularly in the regression framework (Tanaseichuk et al., 2014; Chen et al., 2015; Ning and Beiko, 2015; Wang and Zhao, 2017; Randolph et al., 2018; Xiao et al., 2018). These methods are advantageous over previous methods by taking into account the tree. However, they still have many limitations. For example, some methods do not perform variable selection in model building (Wang and Zhao, 2017; Randolph et al., 2018; Xiao et al., 2018), and hence their prediction performance is subpar for sparse-signal scenarios (i.e., only a subset of OTUs are associated with outcome). For methods that perform variable weighting or selection (Tanaseichuk et al., 2014; Ning and Beiko, 2015), they usually rely solely on the tree topology. The branch lengths, which provide more detailed evolutionary history, are usually ignored. Therefore, there is still a need to develop prediction methods for sparse clustered signals while exploiting the full information of the phylogenetic tree, which consists of both the tree topology and branch lengths.

Previously, we developed *glmgraph* (Chen et al., 2015), a graph-regularized sparse regression model for structured genomic data. In the *glmgraph* framework, besides a sparsity penalty, a graph Laplacian-based structure penalty (Laplacian penalty) was imposed to smooth the coefficients with respect to the graph structure. It also encourages structurally related predictors to be selected simultaneously (Huang et al., 2011). In principle, a graph Laplacian can be constructed based on the pair-wise distances between OTUs with respect to the phylogenetic tree. However, the Laplacian penalty has two major drawbacks for microbiome applications. First, the Laplacian-induced smoothing/grouping effects are susceptible to the interference by a large number of distantly related OTUs since the graph is fully connected. It is well- known that distantly related OTUs have very different biological characteristics, and thus their contribution to the smoothing should be minimized. Second, the smoothing effects induced by the Laplacian penalty is completely driven by the external graph structure. This is in stark contrast to the *l*_2_ penalty-induced smoothing effects (Zou and Hastie, 2005; Huang et al., 2016), which are mainly driven by the internal correlation structure in the data. In case of a misspecified tree, the Laplacian penalty cannot reduce to the *l*_2_ penalty. Therefore, it does not possess the data-driven smoothing property, which has been shown to be important to improve prediction performance under certain scenarios (Waldron et al., 2011).

In this work, in parallel to our previous prediction method for “dense and clustered” microbiome signals (Xiao et al., 2018), we develop a phylogeny-regularized sparse regression model for “sparse and clustered” microbiome signals. The proposed method uses a novel phylogeny-based smoothness penalty, which is defined based on the inverse of the phylogeny-induced correlation matrix. The new penalty addresses the two major drawbacks of the Laplacian penalty: it encourages local smoothing, i.e., smoothing effects from more immediate neighbors, as well as enjoys the data-driven smoothing property if the tree is misspecified. In summary, the sparse nature of the distribution of OTUs in complex microbiome data can be better captured by our model because it provides a data-adaptive way to group the OTUs according to their phylogeny as well as to select the most predictive OTUs, which leads to improved prediction and interpretation.

## 2. Methods

### 2.1. A Phylogeny-Induced Correlation Structure Among OTUs

We first introduce a phylogeny-induced correlation structure, on which our phylogeny-based smoothness penalty will be defined. Suppose we have *p* OTUs on a phylogenetic tree, following the evolutionary model proposed in Martins and Hansen (1997), the correlation of the traits between OTU *i* and *j* can be modeled as

(1) $$c_{ij}(\alpha )=e^{-2\alpha d_{ij}},\;i,j=1,\ldots ,p,$$

where *d*_*ij*_ is the patristic distance between OTU *i* and *j* (i.e., the length of the shortest path linking the two OTUs on the tree) and the parameter α∈(0, ∞) characterizes the evolutionary rate. When α = 0, *c*_*ij*_ = 1 ∀*i, j*, indicating all the traits are the same and there is no evolution. When α → ∞, *c*_*ij*_ = 0 ∀*i*≠*j*, indicating that the traits evolve independently. The parameter α is also related to the phylogenetic depth of trait conservation (Martiny et al., 2015), with a smaller α value indicating a greater phylogenetic depth at which the trait is conserved (i.e., a large clade of OTUs share the trait). In other words, the parameter α has a (soft) grouping effect and groups the OTUs at various phylogenetic depths. Compared to the taxonomic grouping, where the OTUs are grouped at a specific taxonomic level, such phylogeny-based grouping not only achieves more resolutions, but also circumvents the difficulty of the uncertainty in taxonomy assignments. Therefore, in the context of predictive modeling, the parameter α can be treated as a tuning parameter, which allows us to explore different phylogenetic depths to optimize prediction. Also to be noted, the pairwise distance *d*_*ij*_ can be simply the genetic distance based on pairwise comparison of the DNA sequences without the need for explicit tree construction.

### 2.2. Phylogeny-Regularized Sparse Generalized Linear Model

To account for the high dimensionality and the phylogenetic tree structure in microbiome-based prediction, we introduce a phylogeny-regularized sparse generalized linear model. We assume that there are *n* samples with the abundances of *p* OTUs being profiled. For the *i*th sample, let *y*_*i*_ denote the outcome variable, which can be binary or continuous, and ***x_i_*** = (*x*_*i*1_, *x*_*i*2_, … , *x_ip_*)^*T*^ denote the normalized and properly transformed abundance vector of the *p* OTUs. We further assume the data have been standardized ($\underset{i}{\sum }x_{ij}=0,\underset{i}{\sum }x_{ij}^{2}=n$). The goal is to predict *y*_*i*_ based on **x**_*i*_. We will use a generalized linear model

$$g(E(y_{i}))=\beta_{0}+x_{i}^{T}\beta$$

where β_0_ is the intercept, **β** = (β_1_, β_2_, …, β_*p*_) and *g*(.) is a link function (identity and logit link for continuous and binary outcome, respectively). Since *p* > *n*, we need to make some sparsity assumption in order for the model to be estimable. Additional assumption will be imposed on the structural relationship among the model parameters to make the estimation more efficient. To this end, we propose the following penalized log-likelihood to estimate the regression coefficients:

(2) $$pl\left(\beta_{0},\beta ;\lambda_{1},\lambda_{2}\right)=\frac{1}{n}\sum_{i=1}^{n}\left\{-l\left(\beta_{0},\beta ;y_{i},x_{i}\right)\right\}+p_{\lambda_{1}}^{sp}\left(\beta \right)+p_{\lambda_{2}}^{sm}\left(\beta \right),$$

where

$$\begin{array}{l} l(\beta_{0},\beta ;y_{i},x_{i})\\ \;=\{\begin{array}{ll} -{\left(y_{i}-\beta_{0}-x_{i}^{T}\beta \right)}^{2}/2 & \text{ linear regression,}\\ y_{i}\left(\beta_{0}+x_{i}^{T}\beta \right)-\log\left(1+e^{\beta_{0}+x_{i}^{T}\beta }\right) & \text{ logistic regression.} \end{array} \end{array}$$

The penalized likelihood estimate can be obtained by solving the optimization problem

(3) $$\hat{\beta }={\text{argmin}}_{\beta_{0},\beta }pl(\beta_{0},\beta ;\lambda_{1},\lambda_{2}).$$

The two penalty terms in Equation (2) play distinct roles. $p_{\lambda_{1}}^{sp}\left(\beta \right)$ is the sparsity penalty, which induces a sparse solution and has been demonstrated to improve both the prediction performance and model interpretability (Tibshirani, 1996) in the high-dimensional setting. $p_{\lambda_{2}}^{sm}\left(\beta \right)$ is the smoothness penalty, which encourages smoothness of the estimated coefficients with respect to the phylogenetic tree (i.e., encourage similar coefficients for clustered OTUs at a certain phylogenetic depth).

For the sparsity penalty $p_{\lambda_{1}}^{sp}\left(\beta \right)$, we choose to use MCP (Minimax Concave Penalty) (Zhang, 2010):

(4) $$\begin{array}{l} p_{\lambda_{1}}^{sp}(\beta )=\sum_{j=1}^{p}\rho \left(|\beta_{j}|;\lambda_{1},\gamma \right),\;\;\rho \left(t;\lambda_{1},\gamma \right)\\ \;=\lambda_{1}{\int_{0}^{\left|t\right|}(1-x/\left(\gamma \lambda_{1}\right)}_{+}dx, \end{array}$$

where λ_1_ ≥ 0 is the tuning parameter, (.)_+_ indicates the nonnegative part and the parameter γ (1 ≤ γ ≤ +∞) controls the degree of concavity. Larger values of γ make ρ less concave. By varying the value of γ from 1 to +∞, the MCP provides a continuum of penalties with the hard-threshold penalty as γ → 1 and the convex *l*_1_ penalty at γ = +∞. In practice, γ is usually fixed to a reasonable value without the need for further tuning. An important advantage of the MCP over the *l*_1_ penalty is that it leads to a nearly unbiased estimator and achieves selection consistency under weaker conditions. More detailed discussions of MCP could be found in Zhang (2010).

**Figure 1:**
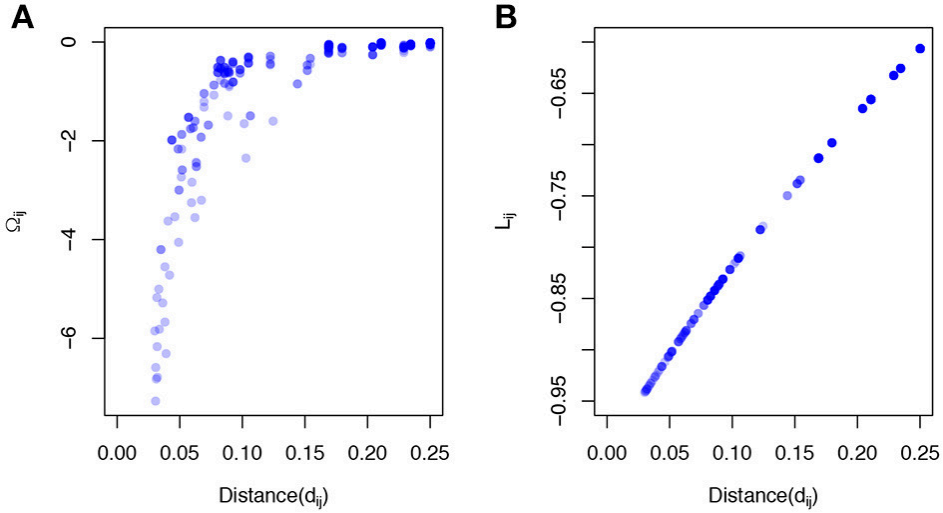
Local smoothing effects of the proposed smoothness penalty. The data was generated based on a simulated phylogenetic tree (*p* = 200,“rcoal” from R “ape” package). The correlation *C*(α) was calculated based on the pairwise patristic distances with α = 2. **(A)** The elements of inverse correlation matrix (Ω_*ij*_) are plotted against pairwise patristic distances (*d*_*ij*_). **(B)** The elements of Laplacian matrix (*L*_*ij*_) are plotted against pairwise patristic distances (*d*_*ij*_).

Our major contribution is the design of a novel structure-based smoothness penalty $p_{\lambda_{2}}^{sm}\left(\beta \right)$ to achieve efficient phylogeny-based smoothing. One common approach to accommodate structure/graph information in sparse regression model is through the use of a graph Laplacian penalty $p_{\lambda_{2}}^{sm}\left(\beta \right)=\lambda_{2}\beta^{T}L\beta$, where the Laplacian matrix *L* is defined based on the connectivity, or adjacency among predictors. The penalized likelihood estimator resulted from the combination of the MCP and Laplacian penalty, termed as *Sparse Laplacian Shrinkage* (SLS) estimator, has been shown to have nice properties such as selection consistency and generalized grouping (Huang et al., 2011). For microbiome applications, a graph Laplacian for microbiome data can be defined using the phylogeny-induced correlation (Equation 1) as the adjacency measure. However, we found that this approach did not always achieve better prediction performance than the procedure without the Laplacian penalty. The subpar performance is partly due to the interference by a large number of distantly related OTUs since the phylogeny-induced graph is fully connected. To achieve better prediction performance, it is important to reduce the contribution of smoothing effects from the large number of distantly related OTUs. Although this can be achieved by sparsifying *L*, in practice, the degree of sparsity to achieve optimal prediction depends on the data and it is difficult to set a universal degree of sparsity for all applications. To overcome the limitation of the graph Laplacian approach, we propose to use an alternative smoothness penalty

(5) $$p_{\lambda_{2}}^{sm}(\beta )=\lambda_{2}\beta^{T}C^{-1}(\alpha )\beta ,$$

where *C*(α) = (_*c*_*ij*_(α))*p* × *p*_ is the phylogeny-induced correlation structure defined in the previous section. The inverse correlation matrix Ω≜*C*^−1^ also implies a graph structure among predictors but encourages more local smoothing, that is, the coefficient smoothing is mainly contributed by its immediate neighbors. To demonstrate a stronger local smoothing effect by Ω than *L*, we plot Ω_*ij*_, *L*_*ij*_, the elements of the Ω and *L*, against the pairwise patristic distances between OTUs (Figure 1). As the pairwise distance increases, Ω_*ij*_ approaches zero quickly while *L*_*ij*_ does not decrease as fast. Since |Ω_*ij*_|, |*L*_*ij*_| determine the contribution of the smoothing effect of OTU *i* to OTU *j*, a faster rate to zero suggests a stronger local smoothing effect.

In the phylogeny-regularized sparse generalized linear model, we have three parameters λ_1_, λ_2_ and α, which need to be tuned in the training step for optimal prediction performance. These three parameters, respectively control the model sparsity (i.e., how many OTUs are predictive of the outcome), the phylogeny-based smoothing effects (i.e., how much smoothing effects should be induced by the tree), and the phylogenetic depth of the signal (i.e., what level of clustering is needed to achieve better prediction). With the inverse correlation matrix-based smoothness penalty, we call the resulting penalized likelihood estimator *Sparse Inverse Correlation Shrinkage* (SICS) estimator. The proposed approach also has a Bayesian interpretation: it assumes that the coefficient **β** has a prior multivariate normal component with the covariance matrix τ*C* and the penalized likelihood estimate can be viewed as the MAP (maximum a posteriori) estimate from a Bayesian perspective.

### 2.3. Connection With Existing Methods

The proposed smoothness penalty **β**^*T*^Ω**β**, the graph Laplacian penalty **β**^*T*^*L***β** and the *l*_2_ penalty **β**^*T*^**β** are all special cases of a general class of quadratic penalties **β**^*T*^Σ**β**, where Σ is a positive semi-definite matrix. When α → ∞, the proposed penalty becomes *l*_2_ penalty and the SICS estimator is reduced to the Mnet estimator (Huang et al., 2016). It is well-known that *l*_2_ penalty induces a grouping effect based on the correlation structure in the data (data-driven smoothing). As α decreases, the phylogeny-driven smoothing will take control (prior-driven smoothing). Thus, α also provides some tradeoff between data-driven and prior-driven smoothing (Theorem 1). To better understand the behavior of the proposed smoothness penalty, we rewrite it as

(6) $$\beta^{T}\Omega \beta =\sum_{i=1}^{p}\left(\Omega_{ii}-\overset{p}{\underset{j=1,j\neq i}{\sum }}|\Omega_{ij}|\right)\beta_{i}^{2}+\sum_{1\leq j<k\leq p}|\Omega_{jk}|{\left(\beta_{j}-s_{jk}\beta_{k}\right)}^{2}$$

where *s*_*jk*_ = sgn(−Ω_*jk*_) is the sign of −Ω_*jk*_. Note that the second part has the same form as the Laplacian penalty (Huang et al., 2011). Thus, the proposed smoothness penalty is a combination of a weighted *l*_2_ penalty (first part) and a Laplacian penalty (second part) with the adjacency coefficients −Ω_*ij*_. For the phylogeny-induced correlation structure, all the off-diagonal elements Ω_*ij*_ are negative and the magnitude controls the prior-driven smoothing effect. The weighted *l*_2_ penalty, on the other hand, offers the data-driven smoothing effect. In contrast, the Laplacian penalty cannot reduce to the *l*_2_ penalty and does not have the data-driven smoothing effect.

Since the proposed smoothness penalty has a weighted *l*_2_ component, some degree of shrinkage in the coefficient estimate is expected (Zou and Hastie, 2005). For orthogonal designs, rescaling could remove the bias due to *l*_2_ shrinkage without significantly increasing the variance. However, we find that, for more general designs, rescaling could instead increase the variance of the SICS estimator and decrease the prediction performance. Therefore, we will not rescale the coefficients in the implementation.

### 2.4. Some Theoretical Properties

We further investigate the smoothing effect and grouping property of the proposed SICS estimator. Previously, Li and Li (2008) derived the smoothing effect and grouping property for the penalty combining *l*_1_ and Laplacian penalty, and Huang et al. (2016) demonstrated a similar property for the Mnet estimator. Here, we demonstrate such property for our SICS estimator under a linear regression model and a simple graph design. The proof of the theorem can be found in the Supplementary File.

Without loss of generality, we assume that the whole graph (as characterized by Ω) corresponding to the index set {1, …, *p*} is divided into disjoint cliques *V*_1_, …, *V*_*J*_. We further assume that the patristic distances between OTUs are the same in each clique so that the phylogeny-induced correlation coefficient *c*_*ij*_ are the same. Thus, Ω has a special block-diagonal structure: Ω = diag(Ω_1_, …, Ω_*J*_) with Ω_*g*_ = (_Ω_*g, lm*_)*v*_*g*_ × *v*_*g*__, where *v*_*g*_ = |*V*_*g*_| for *g* = 1, …, *J*, $\Omega_{g,ll}=\kappa_{g}\left(v_{g}-1\right)\Omega_{g}^{0}$ for Ω_*g*_, κ_*g*_ > 0, *l* = 1, …, *v*_*g*_ and $\Omega_{g,lm}=-\Omega_{g}^{0}$ for 1 ≤ *l, m* ≤ *v*_*j*_, *l*≠*m*. Also, denote $\rho_{jk}=n^{-1}\overset{n}{\underset{i=1}{\sum }}x_{ij}x_{ik}$ (data-induced correlation between OTU *i* and OTU *j*). For the SICS estimator based on this inverse correlation matrix Ω, we have the following smoothing and grouping property:

**Theorem 1**. *Denote* $t=2\lambda_{2}\kappa_{g}\left(v_{g}-1\right)\Omega_{g}^{0}$ *and*

$$\xi =\{\begin{array}{ll} \max\{2\gamma {\left(\gamma t-1\right)}^{-1},(\gamma t+1){\left(t(\gamma t-1)\right)}^{-1},t^{-1}\}, & \text{ if}\;\;\gamma t>1,\\ t^{-1}, & \text{ if}\;\;\gamma t\leq 1. \end{array}$$

Then for *j, k* ∈ *V*_*g*_ and *g* ∈ {1, …, *J*}, we have

$$|{\hat{\beta }}_{j}(\alpha ,\lambda_{1},\lambda_{2})-{\hat{\beta }}_{k}(\alpha ,\lambda_{1},\lambda_{2})|\leq \frac{\xi ||y|{|}_{1}}{\sqrt{n}}\sqrt{2(1-\rho_{jk})}.$$

Especially, if ρ_*jk*_ = 0, we have $|{\hat{\beta }}_{j}\left(\alpha ,\lambda_{1},\lambda_{2}\right)-{\hat{\beta }}_{k}\left(\alpha ,\lambda_{1},\lambda_{2}\right)|\leq \frac{\sqrt{2}\xi ||y|{|}_{1}}{\sqrt{n}}.$

Based on Theorem 1, both the prior-induced correlation *c*_*jk*_ (which in turn determines $\Omega_{g}^{0}$ and ξ) and the data-induced correlation ρ_*jk*_ contribute to the smoothing effect. With the tuning parameter α, *c*_*jk*_ can vary from 0 to 1 (equivalently, $\Omega_{g}^{0}$ varies from 0 to ∞). We can thus increase and decrease the prior-driven smoothing by varying α. The optimal level of prior-driven smoothing effect can be tuned based on the data.

### 2.5. Model Estimation and Computational Complexity

Since the proposed penalty is convex with respect to **β**, coordinate descent algorithm, which is developed for sparse regression model with convex and non-convex sparsity penalties (Friedman et al., 2010; Breheny and Huang, 2011) can be readily extended to our case. For the linear regression model, we have a closed-form solution for each coordinate update. For the logistic regression model, we solve a series of structure-regularized sparse linear regression model at each iteratively reweighed least squares step. Coordinate descent continues until a certain convergence criterion is reached. More details could be found in Chen et al. (2015). We implemented the method in the R package *SICS* (https://github.com/lichen-lab/SICS), which depends on our previously developed *glmgraph* R package (Chen et al., 2015).

The computation complexity of the proposed method consists of two parts: coordinate descent and matrix inversion. For each coordinate descent loop, it requires *O*(*n*+*p*) arithmetic operations, and a full cycle through the *p* OTUs requires *O*(*np*+*p*^2^) operations. Assume the number of iterations to reach convergence is *c*_1_ and the number of tuning parameter combinations is *c*_2_. The overall complexity for the coordinate descent algorithm is thus $O\left(c_{1}c_{2}\left(np+p^{2}\right)\right)$. In addition, taking inverse of the correlation matrix typically has a computational complexity of *O*(*p*^3^) (some algorithm may reduce it, but could not bring down to *O*(*p*^2^)). A total of $O\left(c_{3}p^{3}\right)$ is required to perform matrix inversion, where *c*_3_ is the number of grid points for the tuning parameter α. Therefore, the total computational complexity for SICS is $O\left(c_{1}c_{2}\left(np+p^{2}\right)+c_{3}p^{3}\right)$. Usually, *c*_1_, *c*_2_, *c*_3_ are treated as fixed, so the computational complexity for SICS is *O*(*np*+*p*^3^). Thus it is highly scalable with the sample size but not with the number of OTUs. Since we usually perform OTU filtering before running the algorithm, it is computationally efficient for typical microbiome datasets with *p* < 1000.

## 3. Simulation studies

### 3.1. Simulation Strategy

We performed extensive simulations to evaluate the prediction performance of SICS for both continuous and binary outcome. For the continuous outcome, we simulated 100 samples in the training set and 200 samples in the testing set. For the binary outcome, we simulated an equal number of 50 samples for both case and control groups in the training set, and an equal number of 100 samples in case and control groups in the testing set. We used a Dirichlet-multinomial distribution with parameters estimated from a real microbiome data to simulate OTU counts and generated the outcome based on the abundances of the outcome-associated OTUs. We investigated the effect of the informativeness of the phylogenetic tree and the level of signal strength on the prediction performance. The simulation studies were aimed to reveal the scenarios in which our model performed favorably and also to test whether our model was robust when the phylogenetic tree was not informative or misspecified.

#### 3.1.1. Simulating OTU Abundance Data

We included 200 OTUs in the simulation. The OTU counts were generated using a Dirichlet-multinomial distribution with the parameter values (dispersion, mean proportions) estimated based on a real dataset from the human upper respiratory tract microbiome (Charlson et al., 2010). Only the count data from the 200 most abundant OTUs were used in the parameter estimation. Accordingly, the phylogenetic tree was trimmed to contain the 200 OTUs. For each sample, the total read count was sampled from a negative binomial distribution with mean 5,000 and dispersion 25, reflecting a typical sequencing depth for a targeted sequencing experiment. The OTU counts were normalized into OTU proportions by dividing the total read counts.

#### 3.1.2. Selecting Outcome-Associated OTUs

**Figure 2:**
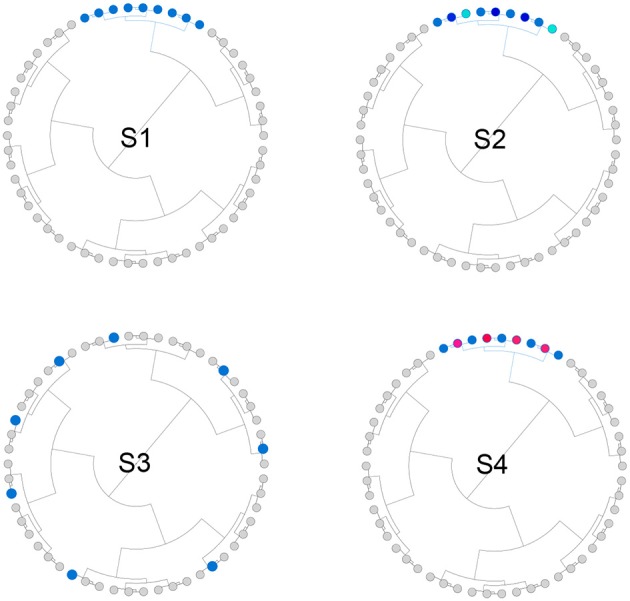
Illustration for the simulation strategy. We simulated both phylogeny-informative scenarios (S1 and S2) and phylogeny-non-informative scenarios (S3 and S4). Blue and red color indicate the direction of the effect while the darkness of the color indicates the magnitude of the effect.

We simulated both phylogeny-informative and non-informative scenarios to study the performance of the proposed method with respect to the informativeness of the phylogenetic tree. In the phylogeny-informative scenarios, we selected outcome-associated OTUs (“aOTUs”) from an OTU cluster and let their effects in the same direction. In the phylogeny-non-informative scenarios, we either randomly selected OTUs or let the effects of the aOTUs in a cluster have opposite effects, which violates the assumption that closely related aOTUs should have similar effects. To construct OTU clusters, we partitioned the 200 OTUs into 20 clusters using the partitioning-around-medoids (PAM) algorithm based on their patristic distances. The simulation strategy was illustrated in Figure 2 and the detailed settings for four scenarios were presented below,

- S1: The phylogenetic tree was informative. One cluster with 12 aOTUs formed an outcome-associated cluster (“aCluster”). In the aCluster, the aOTUs had the same effect size and the effect direction was also the same.
- S2: The phylogenetic tree was informative. On top of S1, we varied the effect size of each aOTU but the effect direction was still the same.
- S3: The phylogenetic tree was non-informative. We randomly selected 12 OTUs to be aOTUs. We restricted one cluster to have only one aOTU.
- S4: The phylogenetic tree was non-informative. On top of S1, we reversed the effect direction for half of the aOTUs.

#### 3.1.3. Generating the Outcome Based on the Outcome-Associated OTUs

Denote A as the set containing the indices of aOTUs, and let *x*_*ij*_ be the proportion of OTU *j* in sample *i*. We first generated η_*i*_ based on the following linear relationship

(7) $$\eta_{i}=\beta_{0}+\sum_{j\in A}\beta_{j}x_{ij}$$

For a continuous outcome,

(8) $$y_{i}=\eta_{i}+\epsilon_{i},\;\;\epsilon_{i}\sim N(0,\sigma_{\epsilon }^{2})$$

For a binary outcome,

(9) $$\begin{array}{l} \;\pi_{i}=\frac{e^{\eta_{i}}}{1+e^{\eta_{i}}}\\ y_{i}\sim Bernoulli(\pi_{i}) \end{array}$$

We simulated different levels of signal strength (effect size). The signal strength was defined as $\frac{\sqrt{\text{var}\left(\eta \right)}}{\sigma_{\epsilon }}$ for the continuous outcome and $\underset{j\in A}{\sum }\text{var}\left({\text{x}}_{j}\right)\beta_{j}^{2}$ (x_*j*_ denotes the abundance for the *j*th OTU) for the binary outcome. In the simulation, we investigated a signal strength at 1.0, 1.5, and 2.0 for continuous outcome and 5.0, 10.0, and 20.0 for binary outcome to represent low, medium and high signal strength. The detailed parameter settings for the four scenarios were included in the Supplementary File.

### 3.2. Competing Methods, Model Selection and Evaluation

#### 3.2.1. Competing Methods

We compared the proposed method (SICS) to Lasso, MCP and Elastic Net (Enet), the three sparse regression models without considering the phylogenetic tree. We also compared SICS to a Laplacian-regularized sparse regression model as implemented in *glmgraph* (SLS) (Chen et al., 2015). The Laplacian matrix *L* was constructed using the same phylogeny-induced correlation matrix *C* as the adjacency matrix. *L* was further sparsified to 90% sparsity level to reduce the adverse effects of distantly related OTUs on the outcome prediction. Besides those sparse regression models, we also compared SICS to a representative machine learning method, Random Forest (RF), which has been demonstrated good prediction performance on microbiome data (Pasolli et al., 2016). The parameter settings for the competing methods were shown in Box 1.

Box 1 **Parameter settings for competing methods**

- Lasso: *glmnet* R package, all parameters were set as the default.
- Elastic Net (Enet): *glmnet* R package. Tuning parameter for *l*_2_ penalty was searched on the grid $\underset{11}{\underset{︸}{\left\{0,0.1,0.2,\cdots \;,1\right\}}}$.
- MCP: *ncvreg* R package, all parameters were set as the default.
- SLS: *glmgraph* R package, the search grid for λ_2_ and α were set the same as SICS.
- Random Forest (RF): *randomForest* R package, parameters were set as default.

#### 3.2.2. Model Selection and Evaluation

For SICS, the parameters (λ_1_, λ_2_, α) were tuned to achieve optimal model sparsity and phylogenetic depth. Specifically, we searched their best combination over a three-dimensional grids. λ_2_ was searched on the grid $\underset{12}{\underset{︸}{\left\{0,2^{-5},2^{-5+\nu },2^{-5+2\nu },\cdots \;,2^{5}\right\}}}$, and α on the grid $\underset{12}{\underset{︸}{\left\{0,2^{-5},2^{-5+\nu },2^{-5+2\nu },\cdots \;,2^{5}\right\}}},\;\nu =1$, while λ_1_ was selected from a finer grid on a log scale from the most sparse to a very dense model as implemented in *glmgraph* and *glmnet*.

The best tuning parameter values were selected based on 5-fold cross-validation (CV), where the training samples were randomly divided into 5-folds with 4-folds for model fitting and the remaining fold for testing . We used PMSE (Predicted Mean Square Error) as the CV criterion for a continuous outcome and AUC (Area Under the Curve) for a binary outcome as in Xiao et al. (2018). Once the optimal tuning parameters were selected, we fit the final model using all the training samples and evaluated the prediction on independent testing samples.

To evaluate the prediction performance, we used PMSE (“Brier score” for a binary outcome), which quantifies the *discrepancy* between the predicted and observed values. In addition, we also investigated the R^2^, which quantifies the (squared) *correlation* between the predicted and observed values and ranges from 0 (no correlation) to 1 (perfect correlation). Detailed definition of R^2^ could be found in Xiao et al. (2018).

Although we focused our evaluation on outcome prediction, variable selection and parameter estimation performance were also investigated to gain more insights about the improved prediction performance of SICS. Variable selection was assessed by sensitivity and specificity, where sensitivity is the true positive rate, i.e., the proportion of aOTUs that are selected, and specificity is true negative rate, i.e., the proportion of irrelevant OTUs that are not selected. The parameter estimation performance was evaluated using MSE (Estimation Mean-Squared Error). Each simulation setting was repeated 50 times and the averages and standard errors of the performance measures were reported.

### 3.3. Simulation Results

#### 3.3.1. Results for Continuous-Outcome Data

**Figure 3:**
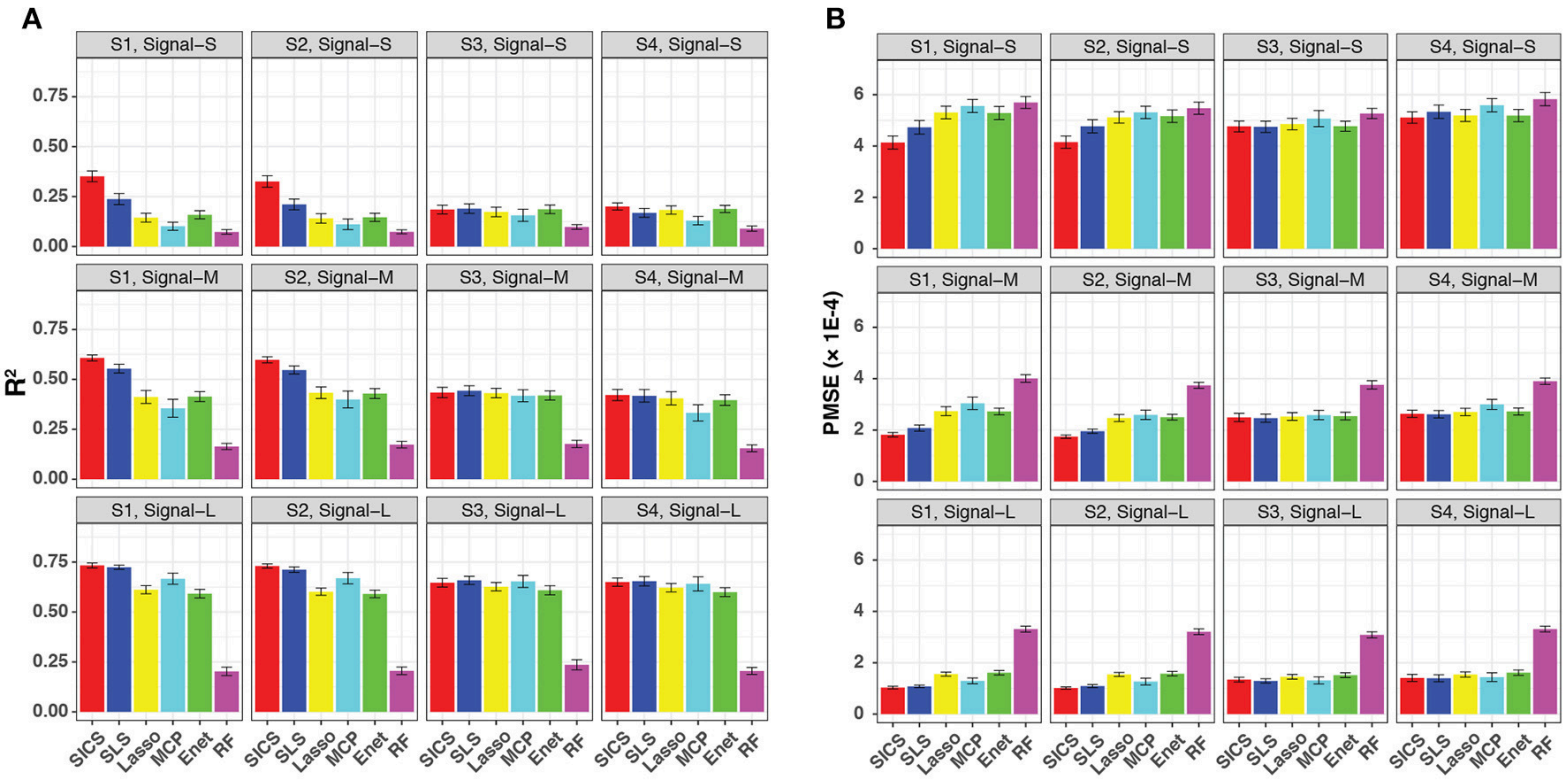
Prediction performance for continuous-outcome simulations across different signal levels and scenarios. Both R^2^ **(A)** and PMSE **(B)** were used for evaluation. S1, S2: phylogeny-informative scenarios, and S3, S4: phylogeny-non-informative scenarios; Signal-S, -M, and -L represent weak, medium and strong signals, respectively.

We evaluated the prediction performance in terms of both R^2^ and PMSE across different scenarios and signal strengths (Figure 3). We observed a general increase in performance for all methods when the signal strength increased. When the phylogenetic tree was informative (Scenario S1 and S2), SICS outperformed other methods substantially with a much larger R^2^ and lower PMSE across all levels of signal strength. The improvement of SICS over other methods was more evident when the signal strength decreased, indicating the importance of using the tree prior to pool signals when the signal was weak. Under the weak signal, SICS had a clear advantage over SLS, which uses the Laplacian penalty to smooth the coefficients, demonstrating the benefit of using the proposed smoothness penalty that encourages more local smoothing. SICS and SLS were both significantly better than other sparse regression methods and RF across different levels of signal strength. The lower performance of these sparse regression methods was due to their inability to exploit the phylogenetic structure. The improved prediction performance of SICS could be explained by more accurate parameter estimation evidenced by a lower MSE (Figure S1) and an increased sensitivity to retain the aOTUs (Figure S2). Although the increased sensitivity was at the cost of a slightly lower specificity (Figure S3), inclusion of aOTUs was more important than exclusion of non-aOTUs to improve prediction. We also observed that SICS performed similarly in Scenario S1, S2, indicating the robustness of SICS to the variation of the effect size of individual aOTUs as long as the effects are in the same direction.

It should be noted that SICS achieved similar performance as other sparse regression methods in its unfavorable scenarios, when the phylogenetic tree was not informative (Scenarios S3 and S4), demonstrating the robustness of SICS. The comparable performance could be explained by that the additional parameters λ_2_, α, which makes MCP and Enet as special cases of SICS.

#### 3.3.2. Results for Binary-Outcome Data

**Figure 4:**
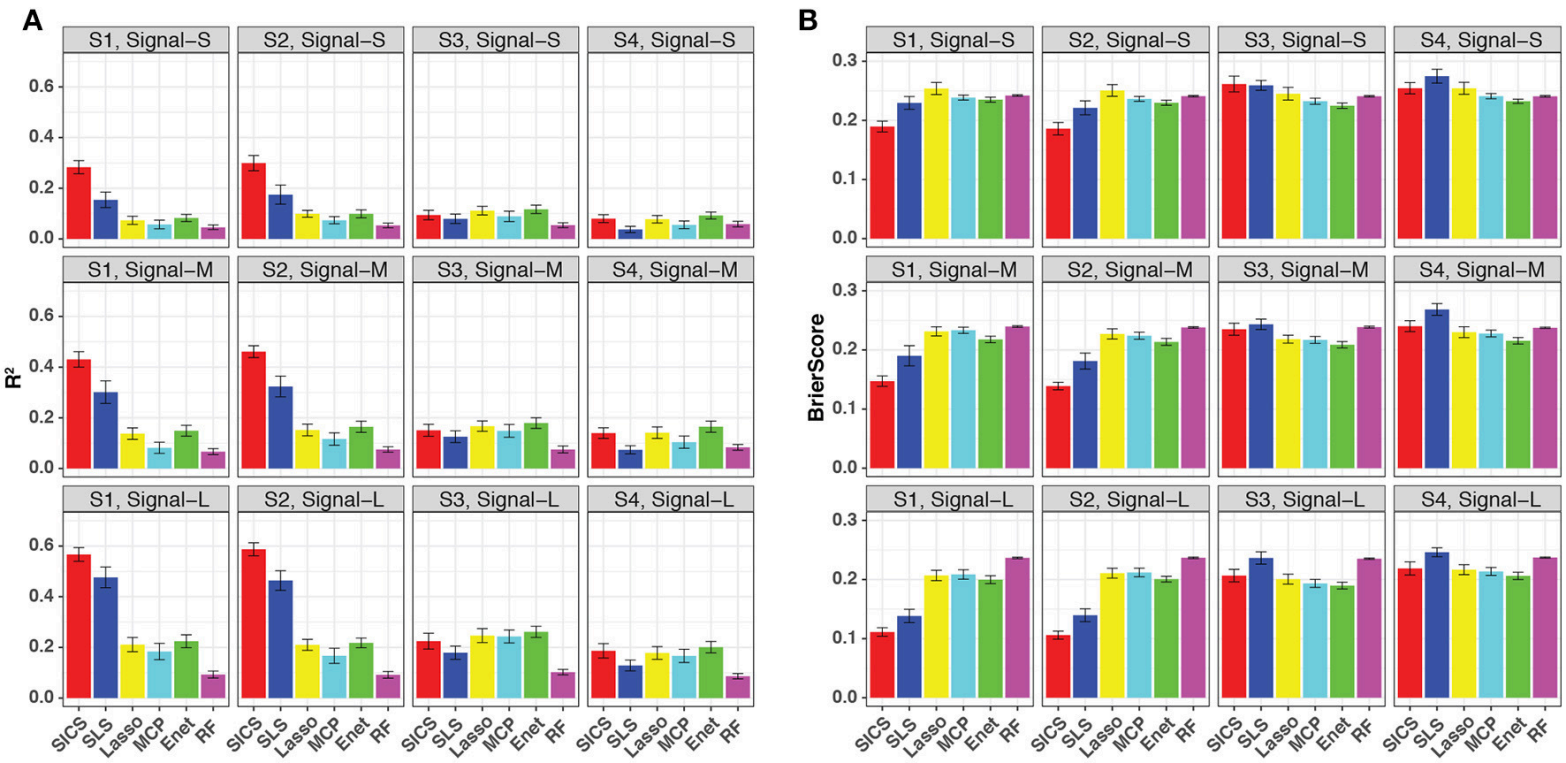
Prediction performance for binary-outcome simulations across different signal levels and scenarios. Both R^2^ **(A)** and Brier score **(B)** were used for evaluation. S1, S2: phylogeny-informative scenarios, and S3, S4: phylogeny-non-informative scenarios; Signal-S, -M, and -L represent weak, medium and strong signals, respectively.

We repeated the same simulations for binary-outcome data and presented the results in Figure 4. Compared to the continuous outcome-based simulations, the prediction improvement of SICS was even more striking when the phylogenetic tree was informative (Scenarios S1 and S2). SICS achieved a significantly larger R^2^ and smaller Brier Score than other methods across different levels of signal strength. The advantage was even evident when the signal was strong, which was not observed for continuous-outcome data. Overall, a similar trend was observed: SICS had the best performance, followed by SLS under an informative phylogeny; SICS was comparable to other methods for a non-informative phylogeny. The advantage of SICS could be explained by a higher sensitivity of selecting aOTUs (Figure S4) at some cost of specificity (Figure S5).

#### 3.3.3. Comparison to SLS With Different Sparsity Levels in the Laplacian Matrix

In the above simulation, we adopted a sparsity level of 90% in the Laplacian matrix *L* for SLS, which generally resulted a satisfactory prediction performance. To further investigate the impact of sparsity level on the prediction performance of SLS, we compared SICS to SLS with different levels of sparsity in *L*. We tested sparsity levels at 0, 10, 30, 50, 70, and 90% and 0% sparsity indicates no sparsification.

For the continuous-outcome data, SICS consistently outperformed SLS in Scenario S1 & S2 when the signal was weak or medium, and was on par with SLS when the signal was strong (Figures S6, S7). When the tree was not informative (Scenarios S3, S4), SLS was not sensitive to the sparsity level as expected and the performance was similar to SICS. For binary-outcome data, the performance difference between SICS and SLS was even more striking and SICS performed much better across levels of signal strength when the phylogeny was informative (Figures S8, S9). We also found that the performance of SLS varied for different levels of sparsity, and SLS generally achieved the best prediction at a sparsity level of 90%. In contrast, SICS did not need to select the optimal sparsity level and had an overall better performance than SLS, regardless of the sparsity level used.

## 4. Real Data Applications

We applied SCIS to two real microbiome datasets and compared it to the competing methods evaluated in the simulations. We compared to two versions of SLS: SLS without sparsifying *L* matrix (SLS(0)) and SLS with 90% sparsity level (SLS(0.9)). In addition, we compared to glmmTree, a phylogeny-regularized linear model for dense and clustered microbiome signals (Xiao et al., 2018). The first dataset came from a study of the impact of the long-term dietary pattern on the gut microbiome. We used the caffein intake as the continuous outcome (Wu et al., 2011). The second dataset came from a study of the smoking effect on the human upper respiratory tract microbiome (Charlson et al., 2010). We used the microbiome data from the left side of the throat and treated the smoking status as the binary outcome.

### 4.1. Caffeine Intake Data

The caffeine intake data was taken from a cross-sectional study of long-term dietary effects on the human gut microbiome in a general population (Wu et al., 2011). The dataset was downloaded from Qiita (https://qiita.ucsd.edu/) with study ID 1011, which consists of 98 samples and 6674 OTUs. We selected the caffeine intake as the outcome of interest since caffeine intake was found to have a significant impact on the gut microbiota (Jaquet et al., 2009). We aimed to predict the caffeine intake based on the OTU abundances. Before applying the prediction methods, we implemented a series of preprocessing steps designed in Xiao et al. (2018) to make the microbiome data more amenable to predictive modeling. First, we removed outlier samples based on an outlier index defined on Bray-Curtis distance and removed rare OTUs with prevalence < 10% to reduce the dimensionality of OTUs, leaving 98 samples and 499 OTUs. Second, we normalized OTU raw read counts using GMPR (Chen et al., 2018) followed by a replacement of outlier counts using winsorization at 97% quantile. Third, we transformed the normalized OTU abundance data using square-root transformation to reduce the influence of highly abundant observation. Finally, we applied quantile transformation to the caffeine intake to make it approximately normally distributed.

**Figure 5:**
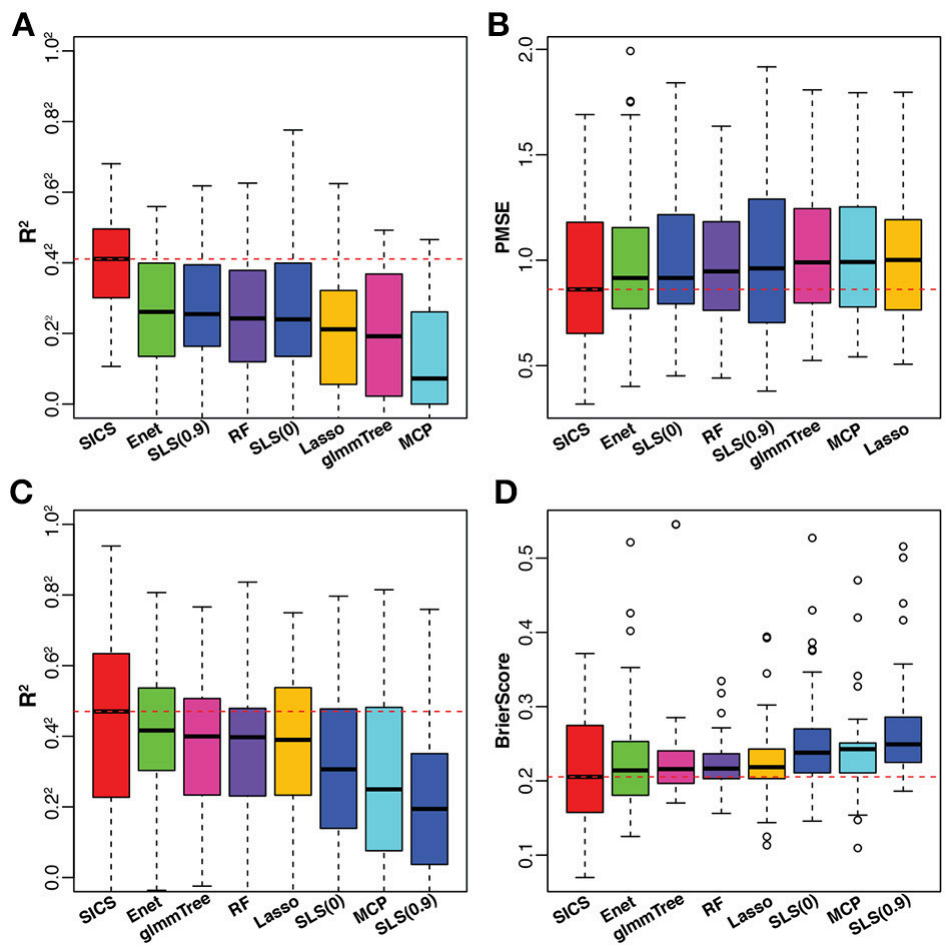
Performance comparison on the caffeine intake data **(A,B)** and smoking data **(C,D)**. The red dashed line indicates the median value of various performance measures for SICS. SLS(0): SLS without sparsification; SLS(0.9): SLS with 90% sparsity level in the Laplacian matrix.

To have an objective evaluation of the prediction performance, the dataset was randomly divided 50 times into 5 folds each time, among which 4 folds were used for training and the remaining one for testing. In the training set, tuning parameter selection was based on CV as in the simulation. R^2^ and PMSE were used as metrics for prediction performance based on the testing set. The results were presented in Figures 5A,B. SICS achieved the best performance for caffeine intake prediction as indicated by the highest R^2^ and lowest PMSE, followed by Elastic Net, SLS and Random Forest. On the other hand, Elastic Net and SLS, which had data-driven smoothing and prior-driven smoothing, respectively, did improve over Lasso and MCP, which only exploited the model sparsity. To verify whether the improvement of prediction was statistically significant, we performed paired Wilcoxon signed-rank test between SICS and any other methods based on R^2^, PMSE values obtained from the fifty random divisions. SICS achieved significantly higher R^2^, and significantly lower PMSE than any other method (*P* < 0.05).

### 4.2. Smoking Data

The smoking data was from a study of the smoking effect on the human upper respiratory tract microbiome (Charlson et al., 2010). We aimed to predict the smoking status based on the microbiome profile. All the data processing steps were carried out as described in the previous example. After preprocessing, the final dataset consisted of 32 non-smokers and 28 smokers with 174 OTUs. For smoking vs. non-smoking prediction, SICS still achieved the highest R^2^ and lowest Brier Score, followed by Elastic Net, glmmTree and Random Forest (Figures 5C,D). However, SLS did not improve the prediction performance compared to Lasso and MCP. We also noticed that SLS(0) and SLS(0.9) performed differently (R^2^ *P* = 0.01; Brier Score *P* = 0.12). Overall, SICS achieved the best prediction performance for both continuous caffeine intake and dichotomous smoking status.

## 5. Discussion

The power of a predictive model depends on its capability to exploit the full information in the data, which usually requires domain knowledge. For microbiome data, one unique characteristic is the phylogenetic relationship relating all OTUs, which is important prior information that could be utilized to improve prediction performance. In this paper, we proposed a phylogeny-regularized sparse regression model for capturing sparse and clustered microbiome signals. In the model, a novel phylogeny-based smoothness penalty was designed based on the inverse of phylogeny-induced correlation matrix. We show that such inverse correlation-based smoothness penalty improved over the traditional Laplacian-based smoothness penalty for microbiome applications, due to its local smoothing property as well as the dual smoothing effects (i.e., data-driven and prior-driven smoothing). Moreover, an additional tuning parameter in the smoothness penalty allows our model to capture signals at various phylogenetic depths, further improving its prediction power. We demonstrated the robustness of the proposed method when the tree was not informative or misspecified. A noisy or misspecified tree could be resulted from applying an inappropriate tree construction method or be due to the fact that DNA sequence similarity does not necessarily reflect biological similarity. Interestingly, the proposed method could reduce to Mnet (Huang et al., 2016), which possesses the data-driven smoothing effect.

Similar to other sparse regression models, the proposed method builds on the assumption that the model is sparse: only a few OTUs are associated with the outcome. It is thus expected to be a powerful predictive tool when the signal is sparse. Many diseases have been shown to be associated with a small number of “marker” taxa. For example, in the case of colorectal cancer or arthritis (Scher et al., 2013; Zeller et al., 2014), individual marker taxa were found to be associated to the disease state, whereas effects on the overall composition were very mild. In contrast, other disease states were associated with marked shifts in the overall composition as in the case of obesity and inflammatory bowel disease (Manichanh et al., 2012; Le Chatelier et al., 2013). In such “dense-signal” scenario, sparse regression models including the proposed approach may not work well. Instead, a prediction model based on the global community similarity, such as our recently proposed glmmTree (Xiao et al., 2018), is expected to be more powerful. Exploratory analysis of the microbiome data should be performed before selecting a suitable model.

In the model, we assume a linear relationship between the OTU abundance and the outcome. Although the assumption is usually reasonable after the abundance data is properly normalized and transformed, it may fail to capture complex nonlinear relationship for some applications. Our model can be extended to capture more complex nonlinear effects. The simplest strategy is to apply various transformations, e.g., Box-cox transformation (Sakia, 1992), to the OTU abundance data and selects the best transformation function based on cross-validation. In the case of Box-cox transformation, the power parameter can be treated as another tuning parameter (Xiao and Chen, 2017; Xiao et al., 2018). Alternatively, one could apply an additive model, which is more flexible and allows OTU-specific nonlinear effects (Wood, 2006). However, a larger sample size may be needed to achieve good performance.

Finally, the distribution of OTU abundances is very skewed, and a large number of OTUs are rare and of low-abundance. For these rare OTUs, their sampling variability is very large. Accommodating the sampling error in the predictive model could potentially improve the prediction performance. Jointly modeling the microbiome and the outcome data is thus a promising direction. We leave these extensions as our future work.

## Author Contributions

JX analyzed the data, wrote the paper, prepared figures and tables, reviewed drafts of the paper. LC analyzed the data, wrote the paper, prepared figures and tables, wrote the software, reviewed drafts of the paper. YY prepared figures and tables, reviewed drafts of the paper. XZ contributed substantial expertise to improve the paper and revised the paper. JC conceived and designed the experiments, analyzed the data, wrote the paper, wrote the software, prepared figures and tables.

### Conflict of Interest Statement

The authors declare that the research was conducted in the absence of any commercial or financial relationships that could be construed as a potential conflict of interest.
