# Supplementary material for: A Phylogeny-Regularized Sparse Regression Model for Predictive Modeling of Microbial Community Data
Source: Front Microbiol. 2018 Dec 19;9:3112. doi: 10.3389/fmicb.2018.03112 (PMC6305753; doi:10.3389/fmicb.2018.03112)
Supplement: Supplementary file 1 [file Data_Sheet_1.pdf]

Supplementary Material: A phylogeny-regularized sparse regression model for predictive modeling of microbial community data

Jian Xiao, Li Chen* and et al.
*Correspondence:
Author Name: Jun Chen, Li Chen
chen.jun2@mayo.edu, li.chen@auburn.edu

1 DETAILS OF SIMULATING SETTINGS

1.1 Continuous outcome

Scenario 1. Set identical coefficients for all casual predictors from one cluster and the coefficients of all the other predictors to 0s. Thus we have

$$\beta^0_{\text{casual}} = \{\underbrace{\beta^0_{\text{casual},1}, \dots, \beta^0_{\text{casual},12}}_{1 \text{ cluster}}\},$$

where $\beta^0_{\text{casual},1} = \cdots = \beta^0_{\text{casual},12} = \frac{6}{\sqrt{10}}$.

Scenario 2. Set similar coefficients for all casual predictors from one cluster and let

$$\beta^0_{\text{casual}} = \{\underbrace{\beta^0_{\text{casual},1}, \dots, \beta^0_{\text{casual},12}}_{1 \text{ cluster}}\},$$

where $\beta^0_{\text{casual,i}} = \frac{4+i}{\sqrt{9}}, i = 1, 2, 3$; $\beta^0_{\text{casual,i}} = \frac{1+i}{\sqrt{10}}, i = 4, 5, 6$; $\beta^0_{\text{casual,i}} = \frac{i-2}{\sqrt{11}}, i = 7, 8, 9$; $\beta^0_{\text{casual,i}} = \frac{i-5}{\sqrt{12}}, i = 10, 11, 12$, and set the coefficients of all the other predictors to 0s.

Scenario 3. Set identical coefficients for all the 12 predictors randomly selected from 12 different clusters. More specifically, let

$$\beta^0_{\text{casual}} = \{\underbrace{\beta^0_{\text{casual},1}, \dots, \beta^0_{\text{casual},12}}_{12 \text{ clusters}}\},$$

where $\beta^0_{\text{casual},1} = \cdots = \beta^0_{\text{casual},12} = \frac{6}{\sqrt{10}}$ and set the coefficients of all the other predictors to 0s.

Scenario 4. Set the OTUs in one cluster to have both positive and negative effects on the outcome. Set

$$\beta^0_{\text{casual}} = \{\underbrace{\beta^0_{\text{casual},1}, \dots, \beta^0_{\text{casual},12}}_{1 \text{ cluster}}\},$$

where $\beta^0_{\text{casual},1} = \cdots = \beta^0_{\text{casual},7} = \frac{6}{\sqrt{10}}$ and $\beta^0_{\text{casual},8} = \cdots = \beta^0_{\text{casual},12} = \frac{-6}{\sqrt{10}}$.

We then adjust σ_ϵ to achieve the desired signal strength.

1.2 Binary outcome

Scenario 1. Set identical coefficients for all casual predictors from one cluster and set the coefficients of all the other predictors to 0s. Given the signal strength SSM = 5.0, 50.0 and 100.0, we let

$$t = \sqrt{\frac{\text{SSM}}{\sum_{j=1}^{12} \text{var}(\text{x}^*_{\text{casual},j})}},$$

and set

$$\beta^0_{\text{casual}} = \underbrace{\{\beta^0_{\text{casual},1}, \cdots, \beta^0_{\text{casual},12}\}}_{\text{1 cluster}},$$

where $\beta^0_{\text{casual},1} = \cdots = \beta^0_{\text{casual},12} = t$.

Scenario 2. Set similar coefficients for all casual predictors from one cluster. Let $w_{\text{casual},i} = \frac{\sqrt{10}}{6} \times \frac{4+i}{\sqrt{9}}, i = 1, 2, 3,\ w_{\text{casual},i} = \frac{\sqrt{10}}{6} \times \frac{1+i}{\sqrt{10}}, i = 4, 5, 6,\ w_{\text{casual},i} = \frac{\sqrt{10}}{6} \times \frac{i-2}{\sqrt{11}}, i = 7, 8, 9,\ w_{\text{casual},i} = \frac{\sqrt{10}}{6} \times \frac{i-5}{\sqrt{12}}, i = 10, 11, 12$, and

$$t = \sqrt{\frac{\text{SSM}}{\sum_{j=1}^{12} w^2_{\text{casual},j}\text{var}(\text{x}^*_{\text{casual},j})}}.$$

We set the coefficients

$$\beta^0_{\text{casual}} = \underbrace{\{\beta^0_{\text{casual},1}, \cdots, \beta^0_{\text{casual},12}\}}_{\text{1 cluster}},$$

where $\beta^0_{\text{casual},i} = w_i * t$ and set the coefficients of all the other predictors to 0s.

Scenario 3. Set identical coefficients for the 12 predictors randomly selected from 12 different clusters. Let

$$t = \sqrt{\frac{\text{SSM}}{\sum_{j=1}^{12} \text{var}(\text{x}^*_{\text{casual},j})}},$$

and set

$$\beta^0_{\text{casual}} = \underbrace{\{\beta^0_{\text{casual},1}, \cdots, \beta^0_{\text{casual},12}\}}_{\text{12 clusters}},$$

where $\beta^0_{\text{casual},1} = \cdots = \beta^0_{\text{casual},12} = t$ and set the coefficients of all the other predictors to be equal to 0.

Scenario 4. Set that OTUs in one cluster can have both positive and negative effects on the outcome. Let

$$t = \sqrt{\frac{\text{SSM}}{\sum_{j=1}^{12} \text{var}(\text{x}^*_{\text{casual},j})}},$$

and set

$$\beta^0_{\text{casual}} = \underbrace{\{\beta^0_{\text{casual},1}, \cdots, \beta^0_{\text{casual},12}\}}_{\text{1 cluster}},$$

where $\beta^0_{\text{casual},1} = \cdots = \beta^0_{\text{casual},7} = t$ and $\beta^0_{\text{casual},8} = \cdots = \beta^0_{\text{casual},12} = -t$.

2 PROOF OF THEOREM 1

Proof. The SICS (sparse inverse correlation matrix shrinkage) estimator can be obtained by

$$\hat{\beta}(\alpha, \lambda_1, \lambda_2) = \arg\min_b$$

$$\{\frac{1}{2n}||\mathrm{y} - \mathrm{X}b||^2 + p^{sp}_{\lambda_1}(\beta) + \frac{1}{2}\lambda_2 \sum_{i=1}^{p}(\Omega_{ii}(\alpha) - \sum_{j=1,j\neq i}^{p} |\Omega_{ij}(\alpha)|)b_i^2 + \frac{1}{2}\lambda_2 \sum_{1\le j<k\le p} |\Omega_{jk}(\alpha)|(b_j - s_{jk}b_k)^2, b \in R^p\}.$$

Then for any $j \in V_g$, $g \in \{1, \ldots, J\}$, the jth estimated coefficient $\hat{\beta}_j(\alpha, \lambda_1, \lambda_2)$ must satisfy the KKT conditions

(I) If $\hat{\beta}_j(\alpha, \lambda_1, \lambda_2) \neq 0$, then

$$-\frac{1}{n}\mathrm{x}_j^T(\mathrm{y} - \mathrm{X}\hat{\beta}(\alpha, \lambda_1, \lambda_2)) + \lambda_1(1 - |\hat{\beta}_j(\alpha, \lambda_1, \lambda_2)|/(\gamma\lambda_1))_+ \mathrm{sgn}(\hat{\beta}_j(\alpha, \lambda_1, \lambda_2))$$

$$+\lambda_2(|\Omega_{g,jj}(\alpha)|\hat{\beta}_j(\alpha, \lambda_1, \lambda_2) - \sum_{k=1,k\neq j}^{v_g} \Omega_{g,jk}(\alpha)\hat{\beta}_k(\alpha, \lambda_1, \lambda_2)) = 0.$$

(II) If $\hat{\beta}_j(\alpha, \lambda_1, \lambda_2) = 0$, then

$$\frac{1}{n}|\mathrm{x}_j^T(\mathrm{y} - \mathrm{X}\hat{\beta}(\alpha, \lambda_1, \lambda_2)) - \lambda_2 \sum_{k=1,k\neq j}^{v_g} \Omega_{g,jk}(\alpha)\hat{\beta}_k(\alpha, \lambda_1, \lambda_2)| \le \lambda_1.$$

Furthermore, let $\hat{r} = \mathrm{y} - \mathrm{X}\hat{\beta}(\alpha, \lambda_1, \lambda_2)$, $\hat{z}_j = \frac{1}{n}\mathrm{x}_j^T\hat{r}$ and $\hat{\Delta}_g = \sum_{k=1}^{v_g} \Omega_{g,jk}(\alpha)\hat{\beta}_k(\alpha, \lambda_1, \lambda_2) = \sum_{k=1}^{v_g}(-\Omega_g^0)\hat{\beta}_k(\alpha, \lambda_1, \lambda_2)$, then (I) and (II) can be written as

(I) If $\hat{\beta}_j(\alpha, \lambda_1, \lambda_2) \neq 0$, then

$$-\gamma\hat{z}_j + (\lambda_1\gamma - |\hat{\beta}_j(\alpha, \lambda_1, \lambda_2)|)_+ \mathrm{sgn}(\hat{\beta}_j(\alpha, \lambda_1, \lambda_2))$$

$$+\lambda_2\gamma(2\kappa_g(v_g - 1)\Omega_g^0\hat{\beta}_j(\alpha, \lambda_1, \lambda_2) - \hat{\Delta}_g) = 0.$$

(II) If $\hat{\beta}_j(\alpha, \lambda_1, \lambda_2) = 0$, then $|\hat{z}_j - \lambda_2\hat{\Delta}_g| \le \lambda_1$.

After some algebra, when $2\lambda_2\gamma\kappa_g(v_g - 1)\Omega_g^0 > 1$, we have

$$\hat{\beta}_j(\alpha, \lambda_1, \lambda_2) = \begin{cases} 0, & \text{if } |\hat{z}_j - \lambda_2\hat{\Delta}_g| \le \lambda_1. \\ \mathrm{sgn}(\hat{z}_j - \lambda_2\hat{\Delta}_g)|\frac{\gamma(|\hat{z}_j - \lambda_2\hat{\Delta}_g| - \lambda_1)}{2\lambda_2\gamma\kappa_g(v_g-1)\Omega_g^0 - 1}|, & \text{if } \lambda_1 < |\hat{z}_j - \lambda_2\hat{\Delta}_g| < 2\lambda_2\gamma\kappa_g(v_g - 1)\Omega_g^0, \\ (2\lambda_2\gamma\kappa_g(v_g - 1)\Omega_g^0)^{-1}(\hat{z}_j - \lambda_2\hat{\Delta}_g), & \text{if } |\hat{z}_j - \lambda_2\hat{\Delta}_g| \ge 2\lambda_1\lambda_2\gamma\kappa_g(v_g - 1)\Omega_g^0, \end{cases}$$

and when $2\lambda_2\gamma\kappa_g(v_g-1)\Omega_g^0 \le 1$, we have

$$\hat{\beta}_j(\alpha,\lambda_1,\lambda_2) = \begin{cases} 0, & \text{if } |\hat{z}_j - \lambda_2\hat{\Delta}_g| \le \lambda_1. \\ (2\lambda_2\gamma\kappa_g(v_g-1)\Omega_g^0)^{-1}(\hat{z}_j - \lambda_2\hat{\Delta}_g), & \text{if } |\hat{z}_j - \lambda_2\hat{\Delta}_g| \ge 2\lambda_1\lambda_2\gamma\kappa_g(v_g-1)\Omega_g^0. \end{cases}$$

First, suppose that feature j and feature k are positively correlated. Based on the above expressions, we can show that

$$|\hat{\beta}_j(\alpha,\lambda_1,\lambda_2) - \hat{\beta}_k(\alpha,\lambda_1,\lambda_2)| \le \xi|\hat{z}_j - \hat{z}_k|,$$

where

$$\xi = \begin{cases} \max\{2\gamma(2\gamma\lambda_2\kappa_g(v_g-1)\Omega_g^0-1)^{-1}, \frac{2\gamma\lambda_2\kappa_g(v_g-1)\Omega_g^0+1}{2\lambda_2\kappa_g(v_g-1)\Omega_g^0(2\gamma\lambda_2\kappa_g(v_g-1)\Omega_g^0-1)}, (2\lambda_2\kappa_g(v_g-1)\Omega_g^0)^{-1}\}, \\ \qquad\qquad\qquad\qquad\qquad\qquad\qquad\qquad\qquad \text{if } 2\gamma\lambda_2\kappa_g(v_g-1)\Omega_g^0 > 1, \\ 2\lambda_2\kappa_g(v_g-1)\Omega_g^0)^{-1}, \qquad\qquad\qquad\qquad\qquad \text{if } 2\gamma\lambda_2\kappa_g(v_g-1)\Omega_g^0 \le 1. \end{cases}$$

By the Cauchy-Schwarz inequality, $|\hat{z}_j - \hat{z}_k| \le \frac{||y||_1}{\sqrt{n}}\sqrt{2(1-\rho_{jk})}$. So we finally get

$$|\hat{\beta}_j(\alpha,\lambda_1,\lambda_2) - \hat{\beta}_k(\alpha,\lambda_1,\lambda_2)| \le \frac{\xi||y||_1}{\sqrt{n}}\sqrt{2(1-\rho_{jk})}.$$

For negative ρ_{jk}, we only need to change the sign of $\hat{z}_k$ and use the same argument. Especially, if $\rho_{jk}=0$, we have $|\hat{\beta}_j(\alpha,\lambda_1,\lambda_2) - \hat{\beta}_k(\alpha,\lambda_1,\lambda_2)| \le \frac{\sqrt{2}\xi||y||_1}{\sqrt{n}}$.

3 SUPPLEMENTARY FIGURES

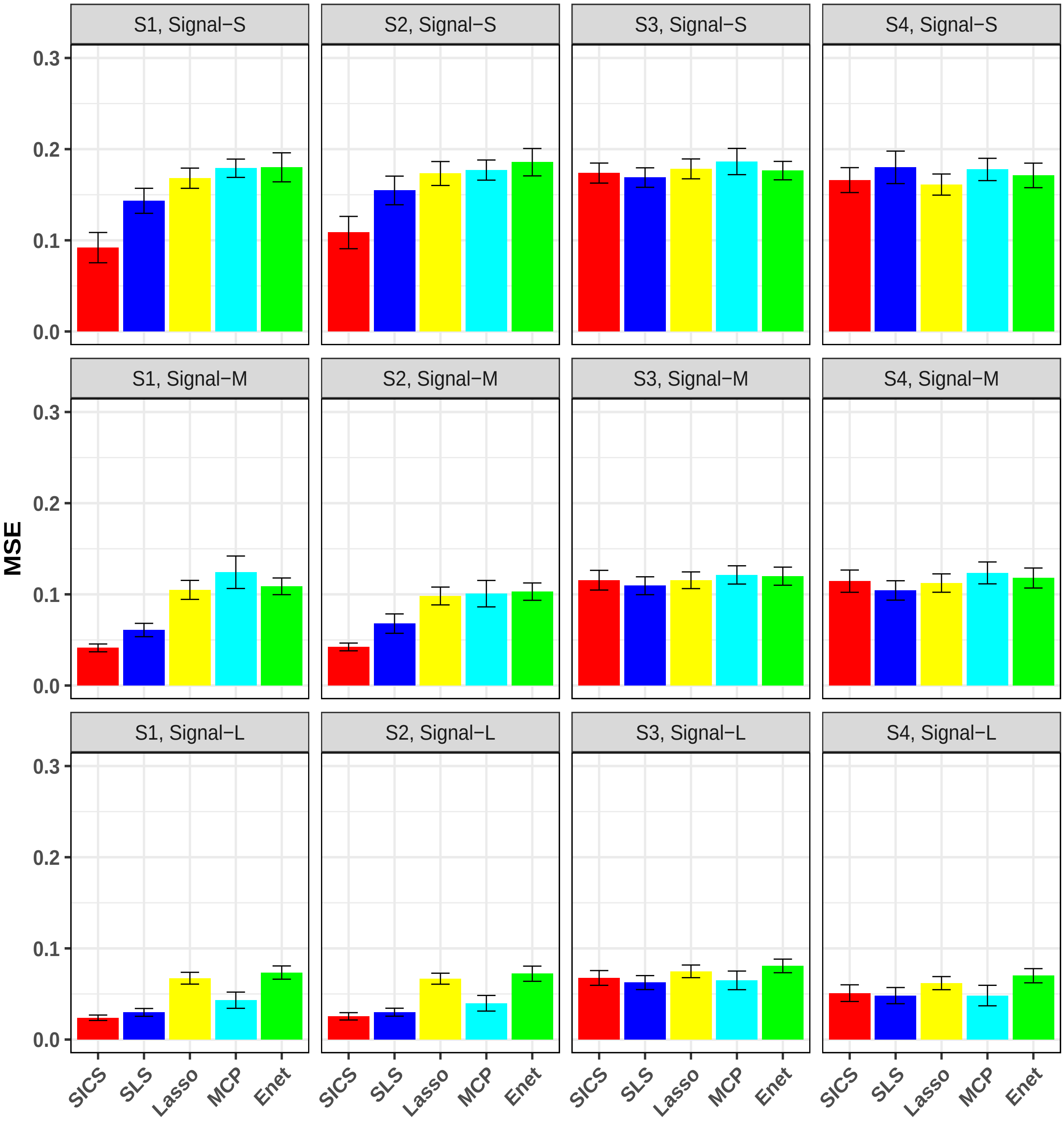

Figure S1. Model estimation performance (MSE) for simulated continuous-outcome data. S1, S2: phylogeny-informative scenarios, and S3, S4: phylogeny-non-informative scenarios; Signal-S, -M, and -L represent weak, medium and strong signals, respectively.

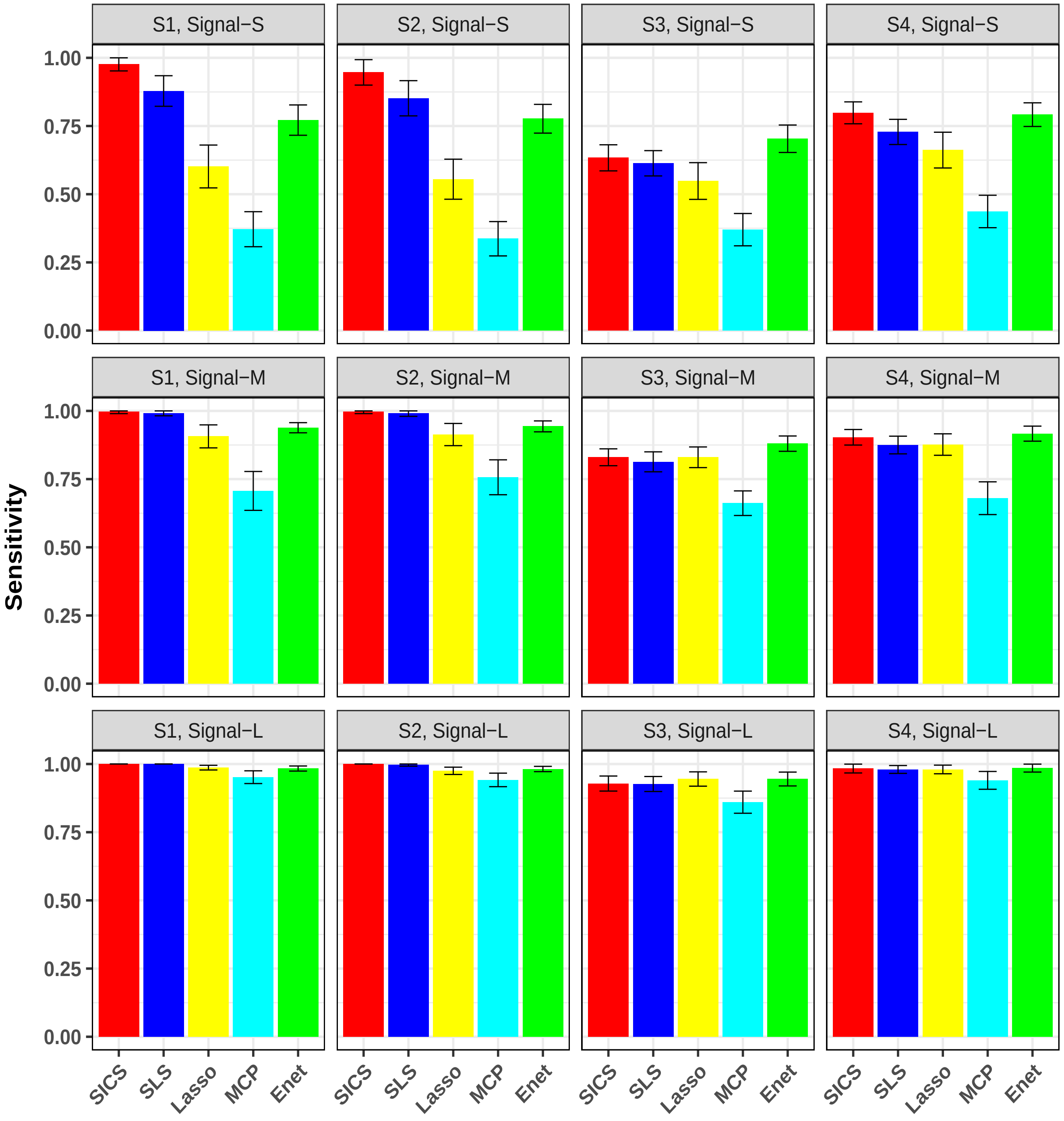

Figure S2. Variable selection performance (Sensitivity) for simulated continuous-outcome data. S1, S2: phylogeny-informative scenarios, and S3, S4: phylogeny-non-informative scenarios; Signal-S, -M, and -L represent weak, medium and strong signals, respectively.

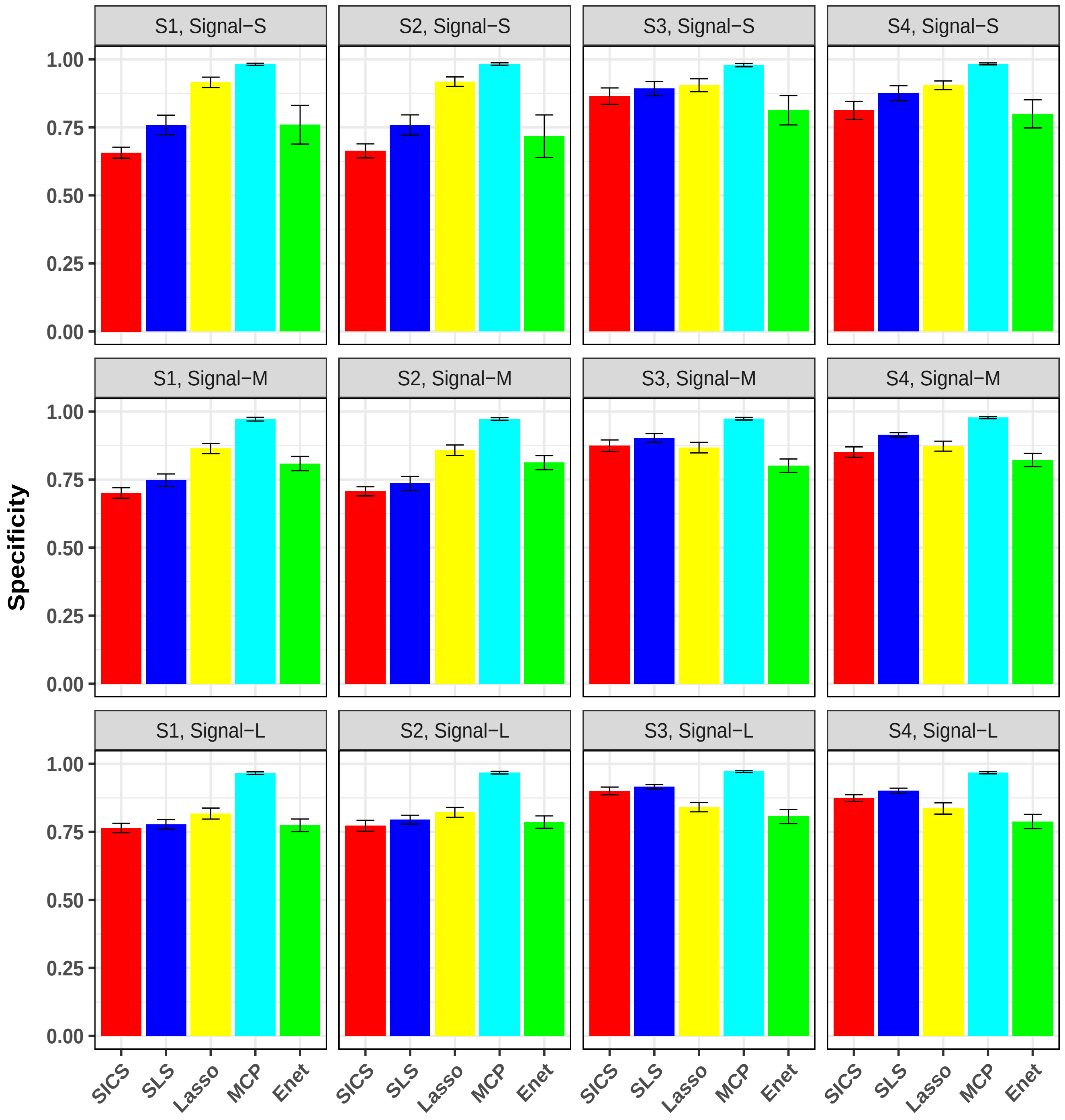

Figure S3. Variable selection performance (Specificity) for simulated continuous-outcome data. S1, S2: phylogeny-informative scenarios, and S3, S4: phylogeny-non-informative scenarios; Signal-S, -M, and -L represent weak, medium and strong signals, respectively.

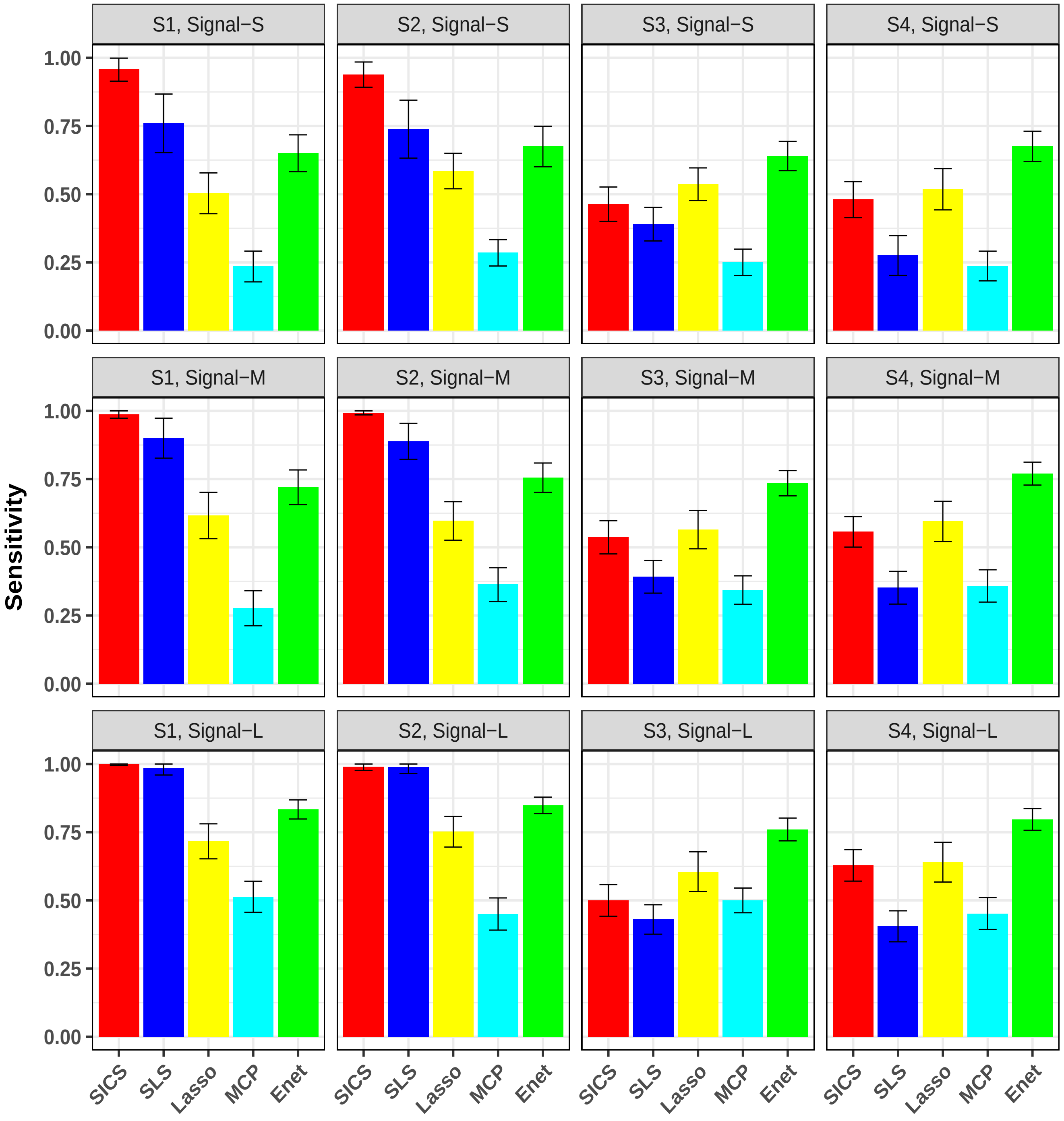

Figure S4. Variable selection performance (Sensitivity) for simulated binary-outcome data. S1, S2: phylogeny-informative scenarios, and S3, S4: phylogeny-non-informative scenarios; Signal-S, -M, and -L represent weak, medium and strong signals, respectively.

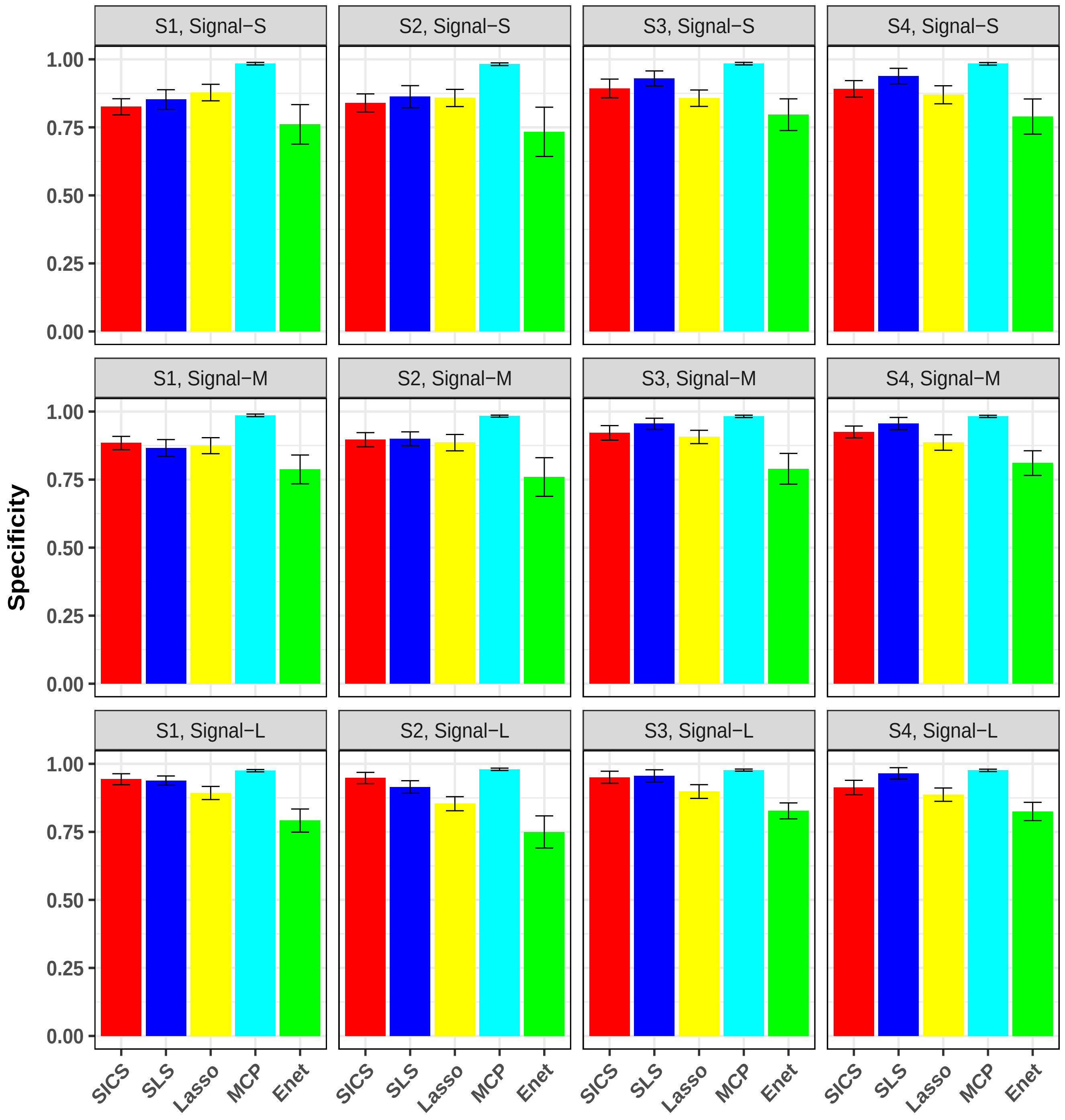

Figure S5. Variable selection performance (Specificity) for simulated continuous-outcome data. S1, S2: phylogeny-informative scenarios, and S3, S4: phylogeny-non-informative scenarios; Signal-S, -M, and -L represent weak, medium and strong signals, respectively.

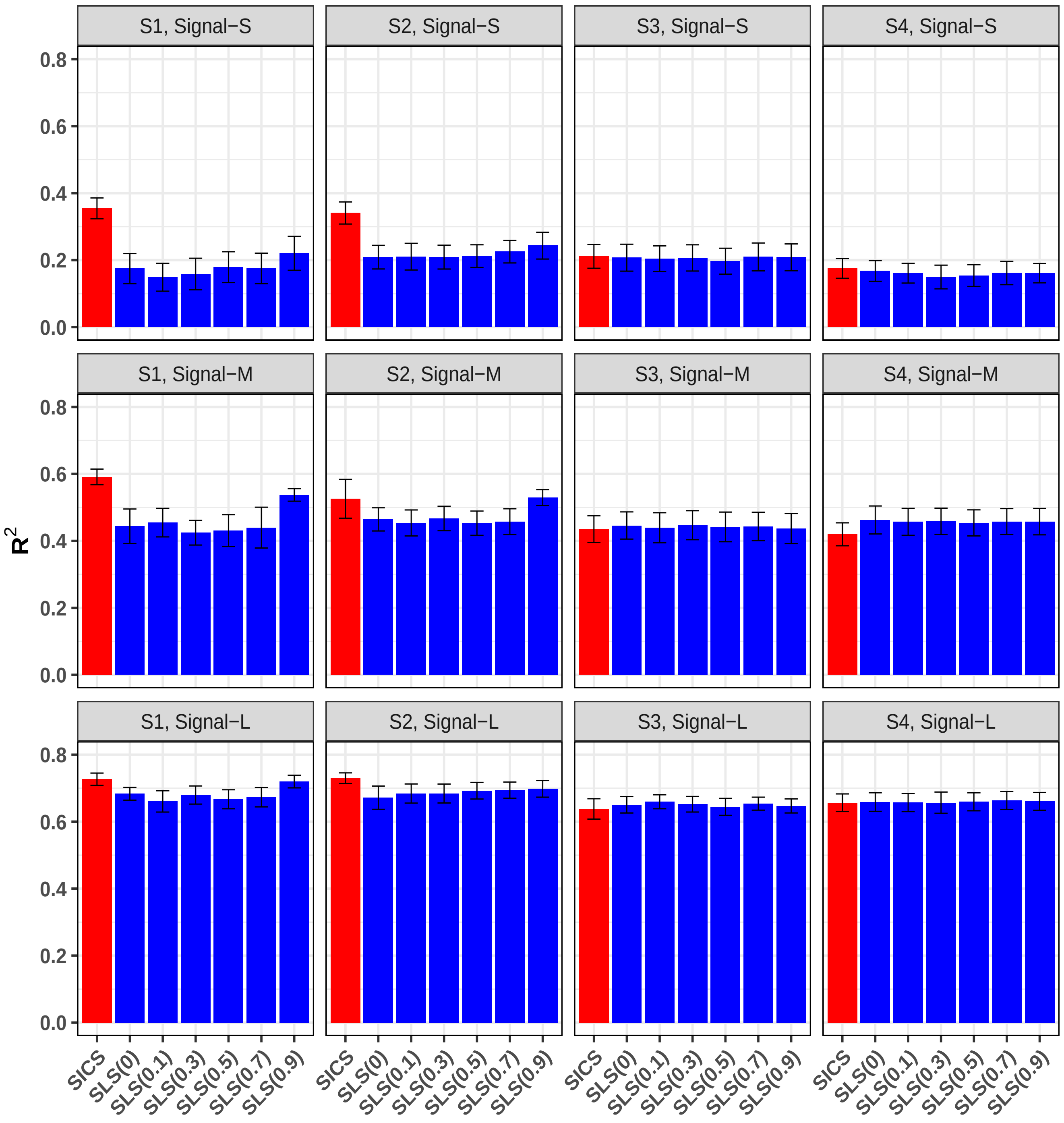

Figure S6. Comparison of SICS to SLS with different sparsity levels in the Laplacian matrix for continuous-outcome data. R^2 was used for evaluating the performance. S1, S2: phylogeny-informative scenarios, and S3, S4: phylogeny-non-informative scenarios; Signal-S, -M, and -L represent weak, medium and strong signals, respectively.

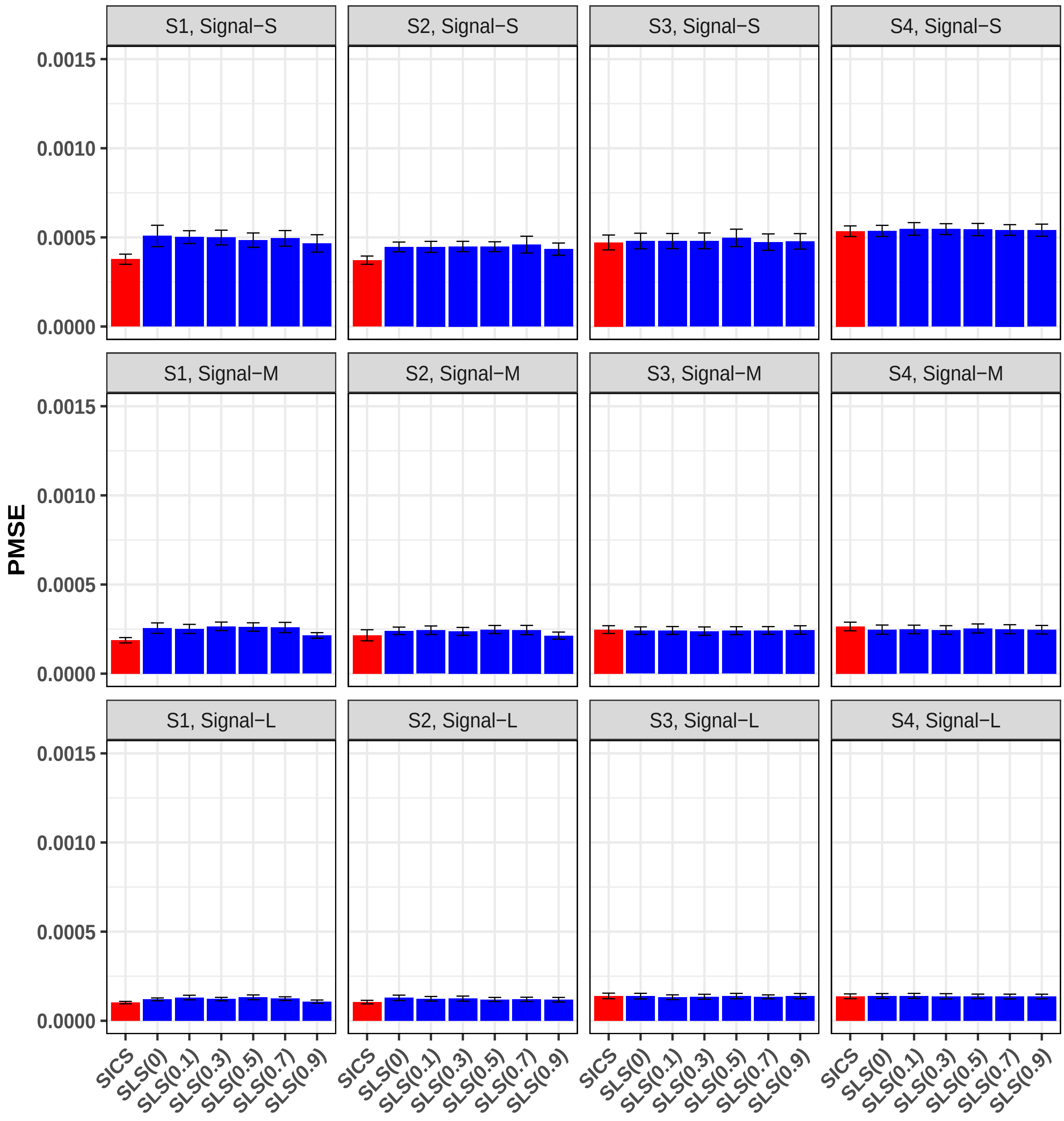

Figure S7. Comparison of SICS to SLS with different sparsity levels in the Laplacian matrix for continuous-outcome data. PMSE was used for evaluating the performance. S1, S2: phylogeny-informative scenarios, and S3, S4: phylogeny-non-informative scenarios; Signal-S, -M, and -L represent weak, medium and strong signals, respectively.

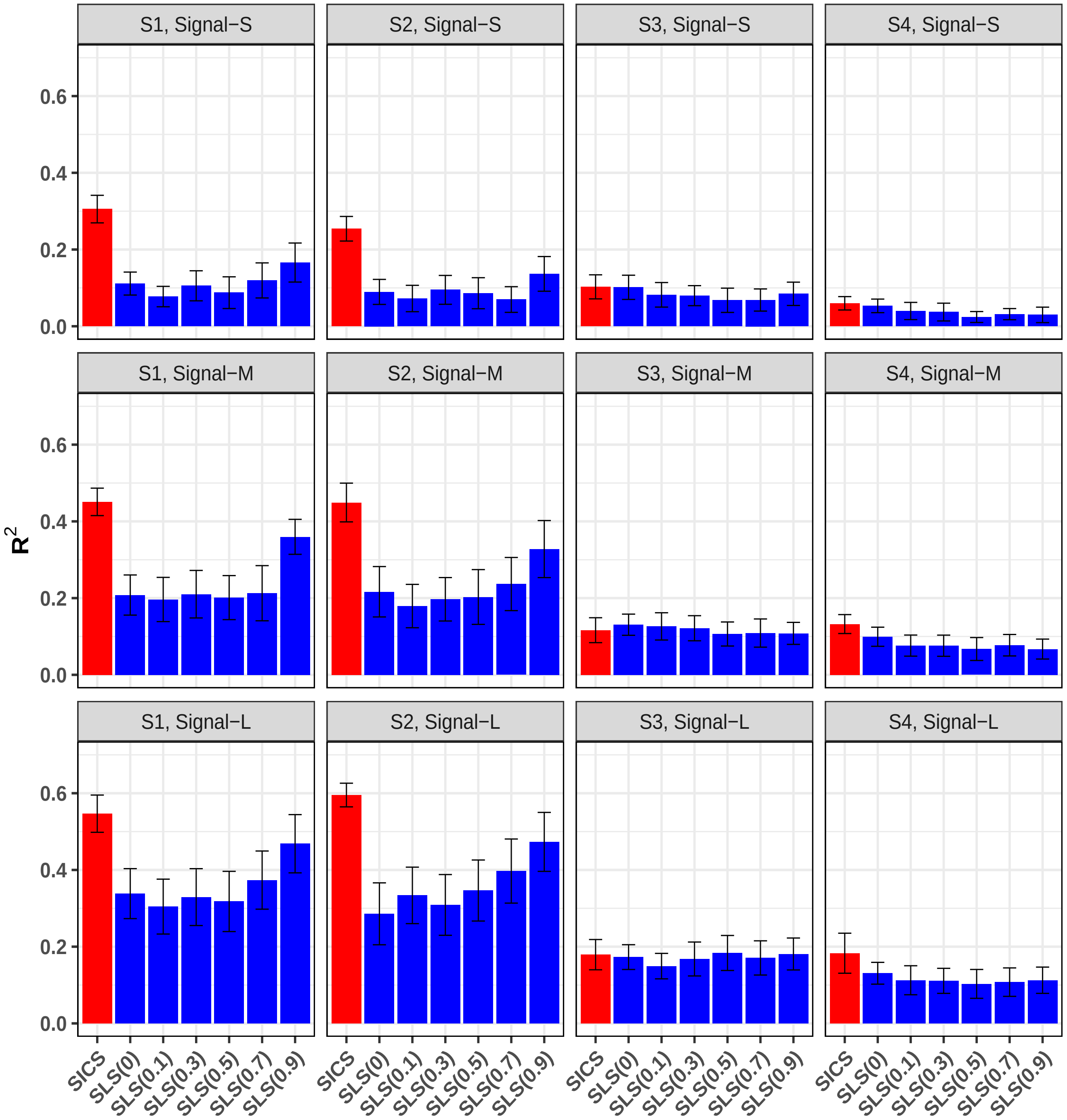

Figure S8. Comparison of SICS to SLS with different sparsity levels in the Laplacian matrix for binary-outcome data. R^2 was used for evaluating the performance. S1, S2: phylogeny-informative scenarios, and S3, S4: phylogeny-non-informative scenarios; Signal-S, -M, and -L represent weak, medium and strong signals, respectively.

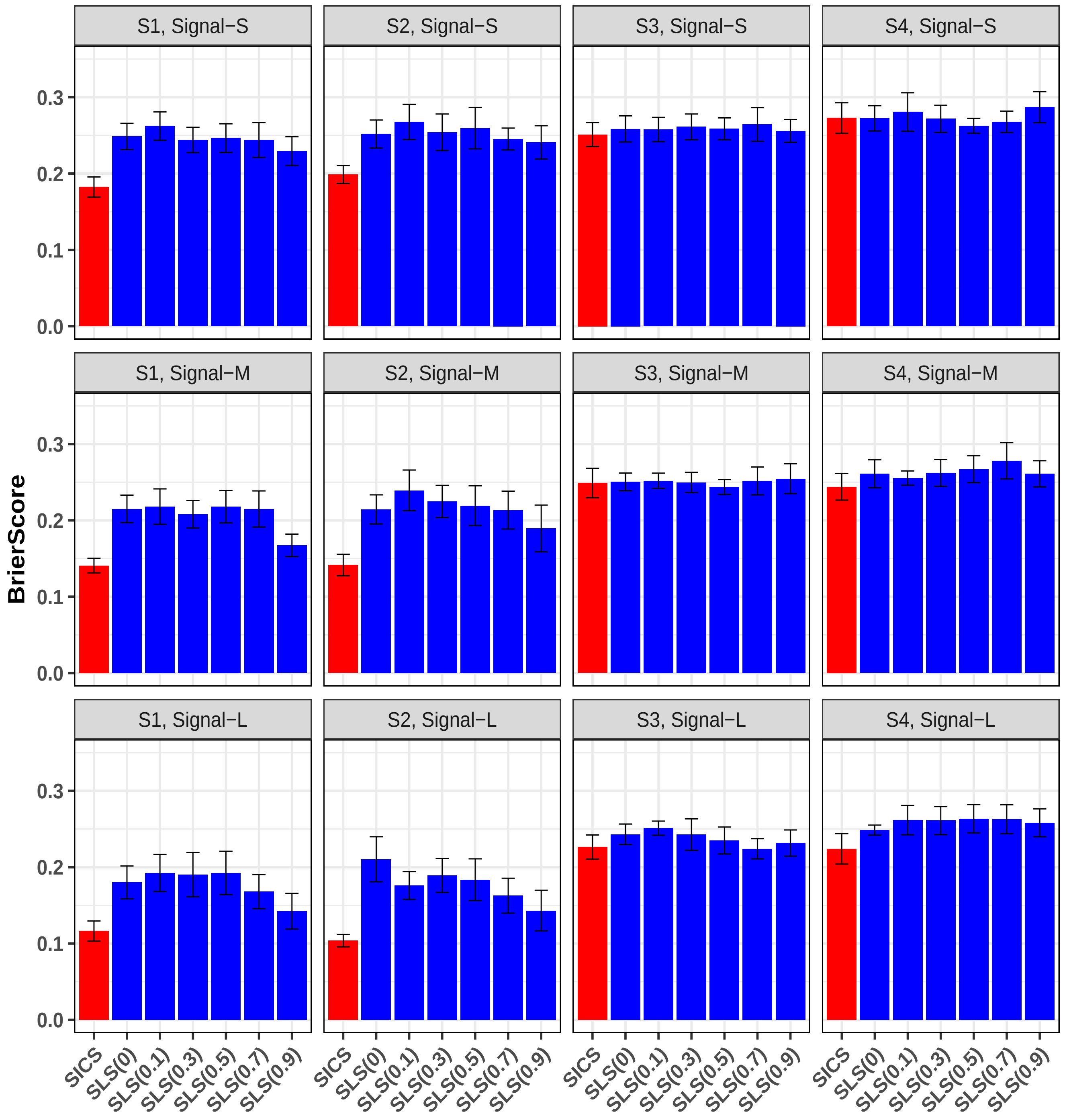

Figure S9. Comparison of SICS to SLS with different sparsity levels in the Laplacian matrix for binary-outcome data. Brier score was used for evaluating the performance. S1, S2: phylogeny-informative scenarios, and S3, S4: phylogeny-non-informative scenarios; Signal-S, -M, and -L represent weak, medium and strong signals, respectively.
